# *Agathobacter rectalis* suppresses colorectal tumorigenesis via an epigallocatechin–SREBF2 axis controlling cholesterol metabolism

**DOI:** 10.1080/19490976.2026.2724227

**Published:** 2026-08-28

**Authors:** Jiawei Lu, Yuting Sun, Yao Zeng, Effie Yin Tung Lau, Zhien Lan, Silin Ye, Rui Zhang, Ruoyu Hu, Chunhui Cui, Jessie Qiaoyi Liang

**Affiliations:** a Department of Medicine and Therapeutics, Li Ka Shing Institute of Health Sciences, CUHK Shenzhen Research Institute, The Chinese University of Hong Kong, Hong Kong, China; b Department of General Surgery, Zhujiang Hospital, Southern Medical University, Guangzhou, China

**Keywords:** *Agathobacter rectalis*, cholesterol metabolism, colorectal cancer, (–)-epigallocatechin, SREBF2

## Abstract

Beneficial effects of the gut commensal Agathobacter rectalis (Ar) are reported in diseases, yet its role in colorectal cancer (CRC) remains unclear. Here, metagenomic analysis revealed consistent fecal Ar depletion across CRC cohorts. In *Apc* ^min/+^ mice, Ar inhibited colon tumorigenesis, reducing tumor number and volume versus E. coli and PBS controls. LC–MS/MS metabolomics showed decreased fecal cholesterol and altered lipid/cholesterol pathways after Ar treatment. *In vitro*, Ar-conditioned medium suppressed CRC cell growth, clonogenicity, migration, and cell cycle progression. LC–MS/MS identified (−)-epigallocatechin (EGC) as an Ar-derived metabolite absent in control bacteria. EGC recapitulated Ar-mediated anti-CRC effects *in vitro* and *in vivo*, with metabolic changes linked to lipid/cholesterol pathways. Transcriptomics showed that EGC suppressed SREBP signaling, cholesterol metabolism, and MAPK pathways. Mechanistically, EGC reduced nuclear SREBF2 and its transcriptional activity, downregulated cholesterol synthesis/metabolism genes, including FDPS and PCSK9, and suppressed MAPK signaling. Molecular docking suggested that EGC may bind pSREBF2 or SCAP. Cellular thermal shift assay revealed that EGC interacts with and stabilizes pSREBF2, but not SCAP. Co-immunoprecipitation demonstrated that EGC reduces pSREBF2–SCAP interaction, thereby inhibiting SCAP-mediated SREBF2 cleavage activation. Together, these findings define an Ar–EGC microbe–metabolite axis and support Ar/EGC-based interventions targeting cholesterol metabolism in CRC.

## Introduction

Colorectal cancer (CRC) is the third most common cancer and the second leading cause of cancer-related death globally.^1^ CRC development is a multifactorial process involving genetic alterations, epigenetic dysregulation, aberrant oncogenic signaling, metabolic reprogramming, immune modulation, and alterations in the gut microbiota. Extensive research focusing on host cells has highlighted complex cancer-related signaling networks and transcriptional and epigenetic regulatory mechanisms contributing to CRC and identified potential new therapeutic avenues.^2^ ^-^ ^5^ On the other hand, accumulating evidence also indicates that the gut microbiota plays an important role in CRC initiation, progression, and therapeutic response.^6^ CRC-associated dysbiosis is characterized not only by enrichment of potentially pro-tumorigenic bacteria, but also by depletion of beneficial commensals, particularly short-chain fatty acid (SCFA)-producing species.^7^ The gut microbiota can promote or suppress colorectal tumorigenesis through multiple mechanisms, including modulation of intestinal barrier integrity, chronic inflammation, host immune responses, microbial colonization resistance, genotoxic stress, and functional metabolite production.^8^ In particular, microbiota-derived metabolites, such as SCFAs, bile acids, tryptophan-derived metabolites, and other bioactive small molecules, have been increasingly recognized as important mediators linking microbial alterations to tumor cell proliferation, immune regulation, and the tumor microenvironment.^9^ ^,^ ^10^

The effectiveness of probiotics in reducing cancer incidence or suppressing tumor growth has been demonstrated in animal models.^11^ ^,^ ^12^ *Agathobacter rectalis* (Ar), previously known as *Eubacterium rectale*, ^13^ is a well-known SCFAs/butyrate-producing bacterium and one of the most abundant bacteria in human gut microbiota. The reduction of fecal Ar has been shown to be associated with multiple diseases, such as intestinal lymphoma and COVID-19,^14^ ^,^ ^15^ and reduced Ar was associated with COVID-19 mortality.^16^ Ar administration attenuated the systemic inflammation induced by herpes simplex virus type 1 in mice.^17^ Beneficial effects of Ar against cancers have also been reported. Ar suppressed lymphomagenesis by alleviating the TNF-induced TLR4/MyD88/NF-κB axis.^14^ Ar also helped improve the efficacy of anti-PD1 immunotherapy in melanoma.^18^ According to these findings, Ar is a promising probiotic candidate for the prevention and management of diseases including cancer. However, its role in CRC remains unclear.

In this study, using our strain (isolated from a healthy volunteer) and strain A1-86 (from DSMZ, also isolated from a healthy volunteer),^19^ we obtained consistent *in vitro* and *in vivo* results demonstrating the tumor-suppressive roles of Ar in CRC. Moreover, we identified a novel anti-tumor metabolite contributing to Ar-mediated tumor suppression and elucidated its underlying mechanism.

## Methods

### Sample processing and metagenome data analysis

The study protocol was approved by the Joint Chinese University of Hong Kong—New Territories East Cluster Clinical Research Ethics Committee (Joint CUHK-NTEC CREC; Ref. No. 2023.328), and written informed consent was obtained from all participants. A total of 276 fecal samples were collected from Hong Kong Chinese participants, comprising 111 patients with colorectal cancer (CRC) and 165 healthy controls. Metagenomic sequencing data from Hong Kong Chinese subjects, Chinese cohort-2 (74 CRC and 54 controls),^20^ French cohort (61 CRC and 53 controls)^21^ and Indian cohort (30 CRC and 30 controls)^22^ were analyzed. The relative abundances of bacterial species were determined by MetaPhlAn4.^23^

### Bacterial culture conditions

*Agathobacter rectalis* (A1-86) was sourced from DSMZ (Braunschweig, Germany). A strain isolated in-house was obtained from the fecal sample of a healthy donor. *Escherichia coli* (MG1655) and *Faecalibacterium prausnitzii* (ATCC 27768) were acquired from the American Type Culture Collection (ATCC; Manassas, VA). Ar and Fp were cultured in Yeast Casitone Fatty Acid (YCFA) medium prepared as previously described,^24^ and *E. coli* was grown in Reinforced Clostridium Medium (Hopebiol, Qingdao, China). All cultures were maintained anaerobically at 37°C. The supernatants were sterilized by filtration through 0.22 μm syringe filters and stored at −80°C until use.

### Animal models

Animal experiments were conducted in accordance with the guidelines approved by the Animal Ethics Committee of The Chinese University of Hong Kong (AEEC ref# 25-096-GRF). *Apc* ^min/+^ C57BL/6J mice (RRID: MGI:2160693, Jackson Laboratory, Bar Harbor, ME), bacterial strains Ar (A1-86), our strain Ar (cuhk280), and *E. coli* MG1655 were used in this study. EGC was purchased from Sigma-Aldrich. Mice were orally gavaged with 10^8^CFU of Ar, E. coli, 100 μM EGC (200 μL), or PBS (200 μL) daily for 12 weeks (*Apc* ^min/+^) or 8 weeks (wild-type). Fecal samples were collected weekly. Colons and sera were collected after mice were sacrificed. All tissues were immediately stored at −80°C or fixed in 4% paraformaldehyde.

### Metabolomics data analysis

Primary data were filtered based on the relative standard deviation and normalized using an internal standard. The final dataset, containing peak numbers, sample names, and normalized peak areas, was imported into the SIMCA 16.0.2 software package (Sartorius Stedim Data Analytics AB, Umea, Sweden) for multivariate analysis. The data were scaled and logarithmically transformed to minimize noise and variance. Afterwards, principal component analysis (PCA) was performed to visualize the sample distribution and grouping. A volcano plot was used to visualize the results of differential metabolite screening in the form. Metabolic pathway analysis based on differentially abundant metabolites was performed using MetaboAnalyst 6.0 (https://www.metaboanalyst.ca/).^25^ Significantly altered pathways were identified by high -log(p) values and high impact values.

### Cholesterol quantification assay

Cholesterol in feces was measured using a commercial kit (BC1985, Solarbio, Beijing, China). Briefly, 30 mg feces was homogenized in 400 μL extraction solution, sonicated on ice, and centrifuged at 10,000 × g and 4°C for 10 min. The supernatant was collected for analysis. Cholesterol standards were prepared by serial dilution. After reaction with working solution at 37°C for 30 min, absorbance was read at 500 nm and cholesterol content was calculated from the standard curve.

### Patient-derived organoids

Patient-derived CRC organoids were seeded in Matrigel (Absin, Shanghai, China) in 24-well plates and maintained at 37°C with 5% CO_2_. After 48 hours, the cultures were treated with either PBS (control) or 160 μM EGC. Images of the same field of view were captured on days 0, 3, and 6, and organoid surface areas were quantified using ImageJ software. Following fixation in 4% paraformaldehyde for 2 hours, the organoids were carefully collected and stabilized within Hitogel (Epredia, Michigan) for further routine IHC staining.

### Histochemistry and Immunohistochemistry

Mouse colon tissues were fixed in 4% paraformaldehyde, paraffin-embedded, and sectioned at 4 μm. Histological evaluation was performed after hematoxylin and eosin staining by a pathologist blinded to treatment groups. Organoids were fixed in 4% paraformaldehyde for 2 h, embedded in Hitogel (Epredia, Michigan) and paraffin, and sectioned at 3 μm. Primary antibody binding was detected using the IHC Select HRP/DAB kit (DAB150, Millipore, Billerica, MA). Positive staining in randomly selected fields was quantified with ImageJ 13.0.6 by two independent investigators.

### Protein extraction and western blotting

Cells were lysed using RIPA lysis buffer (P0013B, Beyotime) containing 1 × protease inhibitor (P1046, Beyotime). The protein lysates were resolved on SDS-PAGE gels and transferred onto a 0.2-μm polyvinylidene fluoride transmembrane for 1.5 h. The membrane was blocked with 5% skimmed milk in TBST solution at room temperature for 1 hour. The membrane was then washed three times with PBST and then incubated at 4°C overnight with a primary antibody. After that, the membrane was washed three times with PBST, incubated at room temperature with the corresponding secondary antibody, and exposed using the Chemidoc MP Imaging system (Bio-Rad, Hercules, CA).

### SREBF2 transcriptional activity assay

SREBF2 transcriptional activity was measured using a CTFB assay according to the manufacturer's instructions. Samples containing SREBP-2 were added to assay wells and incubated overnight at 4°C. After washing, wells were sequentially incubated with primary antibody and horseradish peroxidase-conjugated secondary antibody, followed by color development and reaction termination. Absorbance was measured at 450 nm.

### Molecular docking analysis

Protein structures of SCAP and SREBF2 were obtained from UniProt, and the EGC structure was downloaded from PubChem. After preparation in AutoDockTools, molecular docking was performed using AutoDock Vina (version 1.5.7). The lowest-energy binding pose for each target was selected for analysis. Protein–ligand interactions were visualized using PyMOL (version 4.6) and BIOVIA Discovery Studio (version 2019).

### Cellular thermal shift assay (CETSA)

For CETSA, cells were lysed by freeze–thaw cycles in PBS containing protease inhibitors and centrifuged to obtain soluble fractions. Lysates were incubated with EGC or vehicle for 30 min, heated at the indicated temperatures for 3 min, cooled for 3 min, and centrifuged at 20,000 × g for 20 min at 4°C. Soluble proteins in the supernatants were analyzed by SDS–PAGE and Western blotting.

### Co-Immunoprecipitation (Co-IP)

Co-immunoprecipitation was performed using a commercial kit (HY-K0202K, MedChemExpress) according to the manufacturer's instructions. Cell lysates were incubated with anti-SCAP antibody (ab125186, Abcam), followed by incubation with magnetic beads. Bound proteins were eluted in SDS sample buffer at 95°C for 5 min and analyzed by SDS–PAGE and immunoblotting.

### Statistical analysis

All values are presented as mean ± standard deviation (SD), mean ± standard error of the mean (SEM), or median (interquartile range (IQR)), as appropriate. Comparisons between two groups were performed using paired t-tests or unpaired nonparametric Mann‒Whitney tests where appropriate. ANOVA was used to compare differences among multiple groups, followed by post hoc analyzes using Tukey's multiple comparisons or tests for linear trends. Statistical analyzes were conducted using IBM SPSS Statistics for Windows (version 22.0; IBM Corp., Armonk, NY), GraphPad Prism 9.4.1 (GraphPad Software Inc., San Diego, CA), or MedCalc Statistical Software version 22.021 (MedCalc Software Ltd, Ostend, Belgium). Statistical significance was defined as a two-sided *P*-value of <0.05.

## Results

### Fecal Ar is decreased in patients with CRC

**Figure 1.**
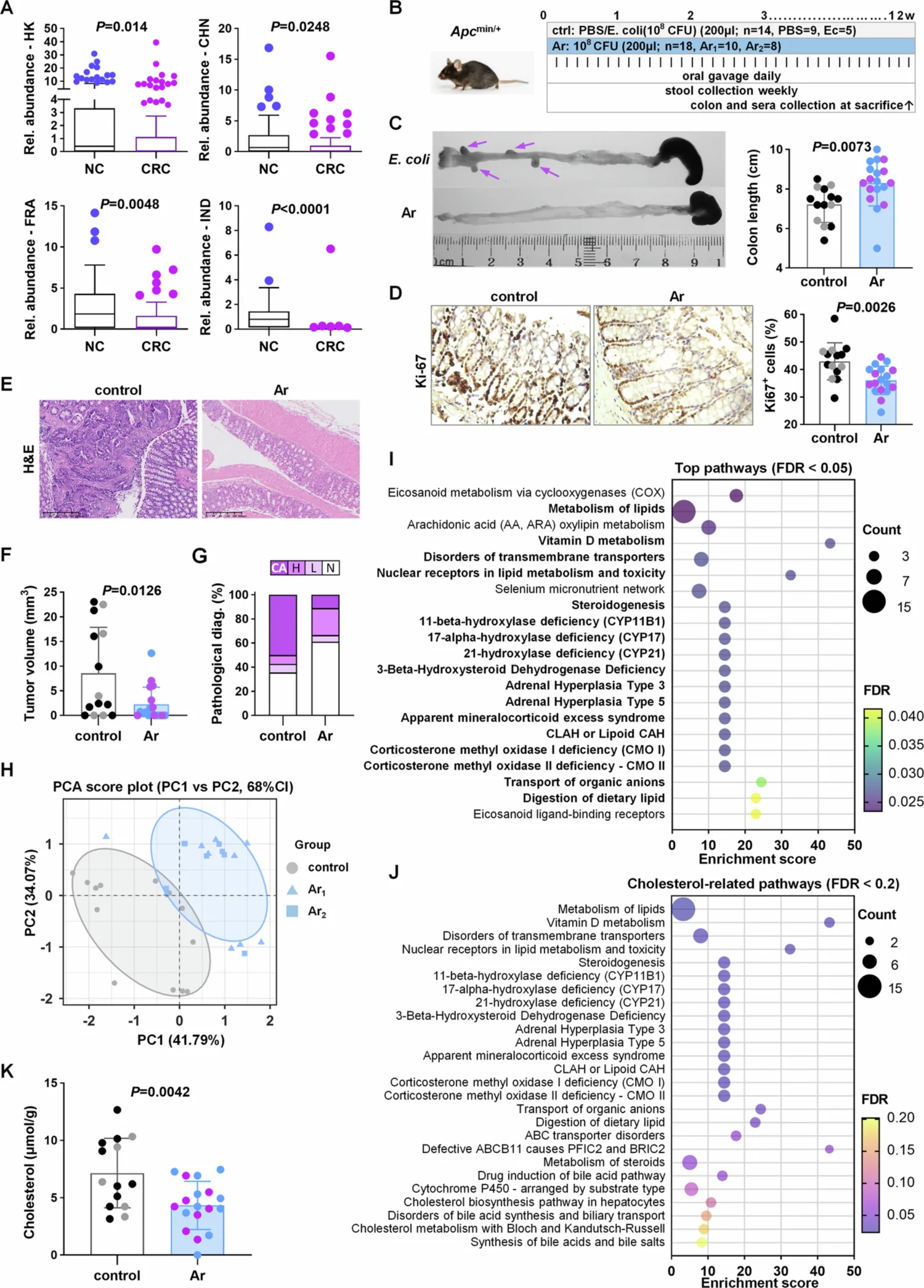
*Agathobacter rectalis* (Ar) is decreased in colorectal cancer (CRC) patients and suppresses colon tumor formation in mice. A. Ar is significantly decreased in feces of CRC patients compared to healthy controls across multiple cohorts. B. Schematic diagram of the Ar treatment plan. C. Representative colon images and comparison of colon length between control and Ar treatment groups. D. Representative images of immunohistochemical Ki-67 staining of non-tumor colon tissues. Ki-67^+^ cells are decreased in Ar treatment groups. E. Representative images of H&E staining of colon tissues. F. Tumor volumes were significantly decreased in Ar-treated groups. G. Comparison of pathological diagnoses of the two treatment groups. CA, cancer; H, high-grade dysplasia; L, low-grade dysplasia; N, normal. H. Scatter plots of principal component analysis (PCA) showing distinct sample distributions between the Ar and control groups along the first two principal components. Ar_1_ refers to our isolated Ar strain; Ar_2_ refers to Ar strain A1-86. I. Most of the top pathways (FDR < 0.05; 17/21) enriched by differential metabolites between Ar and control groups are associated with cholesterol metabolism. J. All identified cholesterol-related pathways enriched by differential metabolites between Ar and control groups (FDR < 0.2). K. Cholesterol quantification assay indicates a significant decrease in cholesterol levels in Ar-treated mice. In panels C, D, F, and K, black dots represent PBS, gray dots represent *E. coli*, blue dots represent our isolated Ar strain, and purple dots represent Ar strain A1-86. As there was no difference between the *E. coli* and PBS groups, or between the two Ar strains, these groups were combined into a single control or Ar group.

By analyzing the metagenomic sequencing data from 111 CRC patients and 165 control subjects, Ar was identified to be significantly decreased in the feces of CRC patients as compared with healthy controls (Figure 1A). The decrease in Ar in CRC was further confirmed in other Chinese and non-Chinese cohorts from published studies.^20^ ^-^ ^22^

### Ar inhibits CRC tumorigenesis *in vivo*

To investigate the effect of Ar *in vivo*, we gavaged Ar (10^8^ CFU) daily to *Apc* ^min/+^ mice, a model with spontaneous CRC and known to exhibit chronic inflammation and increased cellular proliferation in the intestinal crypts, for twelve weeks (Figure 1B). No difference was observed between mice treated with Ar strain A1-86 or our strain isolated from a healthy donor (n = 9 each; Figure S1A-C), so the results were combined. As compared with *E. coli* (non-pathogenic strain) or PBS treatment, the colon lengths of Ar-treated mice were significantly longer (Figure 1C), suggesting improved intestinal health of *Apc* ^min/+^ mice known with chronic inflammation. Ar treatment markedly reduced Ki-67 staining that was elevated in the non-neoplastic intestinal crypts of *Apc* ^min/+^ mice, restoring its localization predominantly to epithelial cells at the crypt base (Figure 1D). Moreover, tumor volume and pathological stages were decreased following Ar treatment (Figure 1E−G). These findings demonstrate a tumor-suppressive role of Ar.

Species-specific qPCR confirmed that fecal Ar abundance was undetectable in control mice but markedly elevated in mice receiving Ar at days 35 and 63 (Figure S1D-E). Metagenomic analysis revealed no significant changes in alpha diversity (Shannon, Simpson, inverse Simpson indices, or observed species richness) following Ar treatment (Figure S1F). Nevertheless, Bray–Curtis distance-based PCoA and PERMANOVA revealed significant differences in microbial community structure between groups (Figure S1G). These results demonstrate that Ar successfully colonizes the gut and reshapes microbial community composition.

### Ar modulates cholesterol metabolism in the gut

We compared fecal metabolomes between the Ar and control groups using untargeted LC–MS/MS; PCA plots revealed clear separation between the groups, indicating distinct metabolic profiles (Figure 1H). Differentially abundant metabolites were subjected to pathway enrichment analysis. Using an FDR threshold of <0.05, 17 of the 21 identified pathways were associated with cholesterol and lipid metabolism (Figure 1I). When expanding the threshold to an FDR < 0.2, 26 pathways related to cholesterol and lipid metabolism were enriched (Figure 1J and Table S1). Pathway-level mapping further showed that Ar treatment affected multiple cholesterol- and lipid-related pathways, including vitamin D metabolism, bile acid metabolism, steroidogenesis, cholesterol biosynthesis, and inflammatory lipid mediator metabolism (Figure S2A), suggesting that Ar treatment broadly remodels cholesterol- and lipid-associated metabolic networks. One metabolite with antitumor properties, macelignan,^26^ was significantly upregulated in the Ar group (Figure S2B), supporting a beneficial role of Ar in modulating gut microbiota metabolism. Consistently, LC–MS/MS indicated reduced cholesterol levels in Ar-treated mice (Figure S2 B-C). We further validated total cholesterol levels in mouse feces using a cholesterol quantification assay. The results showed that, compared with controls, Ar treatment significantly decreased cholesterol levels (Figure 1K and Figure S2D).

### Ar-conditioned medium suppresses CRC cells *in vitro*

**Figure 2.**
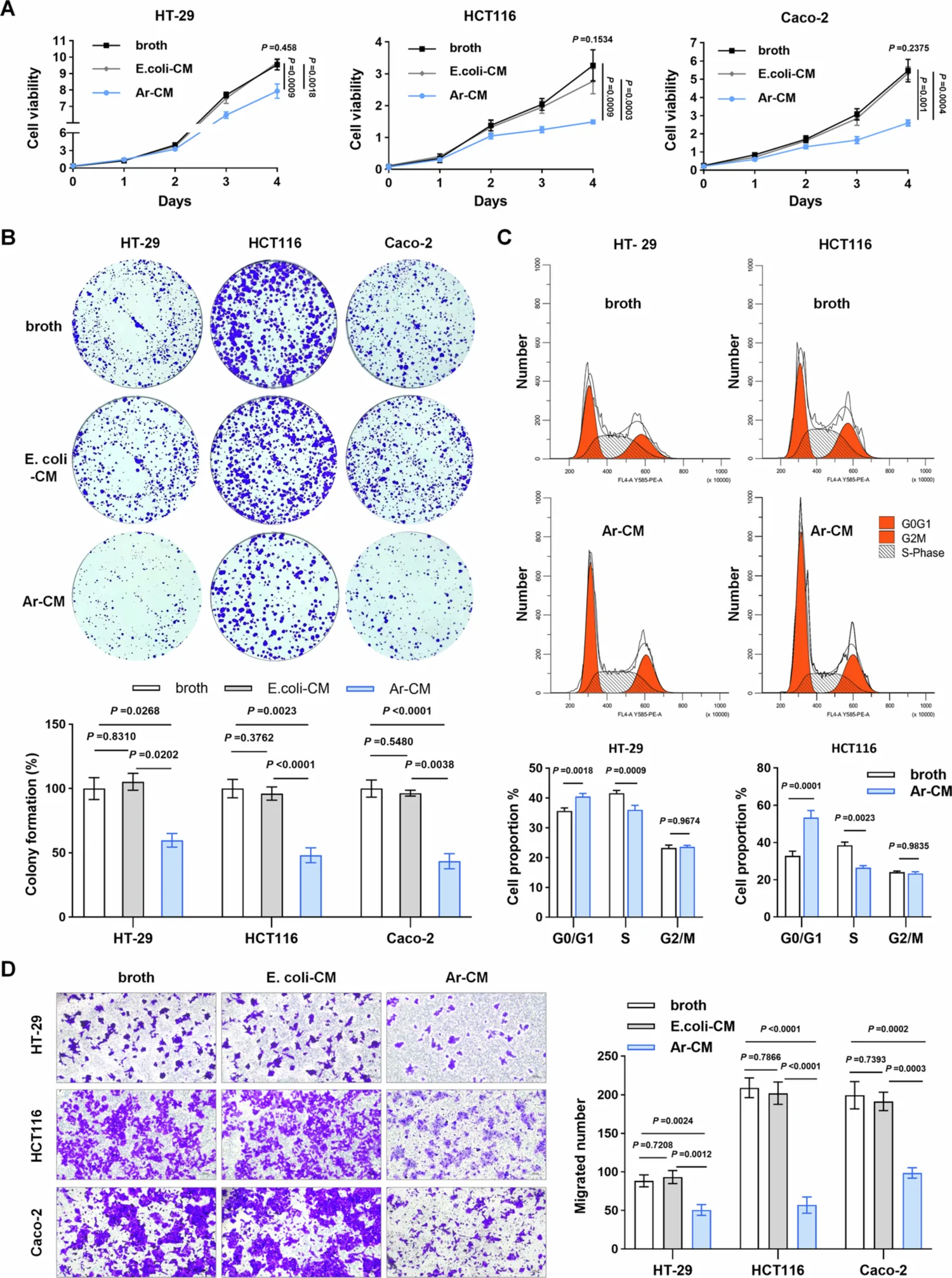
Ar exerts an inhibitory effect on CRC *in vitro*. A. Treatment with Ar-conditioned medium (CM) inhibited the growth of colon cancer cells compared to *E. coli*-CM and broth control treatments. B. Treatment with Ar-CM decreased the colony formation ability of colon cancer cells. C. Treatment with Ar-CM significantly suppressed cell cycle progression. D. Transwell assay revealed that Ar-CM suppressed the migration of colon cancer cells.

To investigate the effects of Ar on cell growth, we treated colon cancer cell lines with Ar-conditioned medium, using *E. coli* (non-pathogenic strain) conditioned medium and broth as controls. Ar-conditioned medium significantly inhibited proliferation and reduced clonogenicity compared to control treatments (Figure 2A–B). Moreover, flow cytometry revealed that Ar-conditioned medium significantly arrested colon cancer cells at the G0/G1 phase and reduced cells in the S phase (Figure 2C), and significantly increased apoptosis in colon cancer cells (Figure S3), indicating that Ar-derived soluble factors suppress CRC cell growth and survival. In addition, transwell assays revealed that Ar-conditioned medium significantly suppressed the migration of colon cancer cells compared to control treatments (Figure 2D). These results indicate that Ar-conditioned medium contains metabolites with anti-CRC effects.

### EGC is a potential functional metabolite of Ar

**Figure 3.**
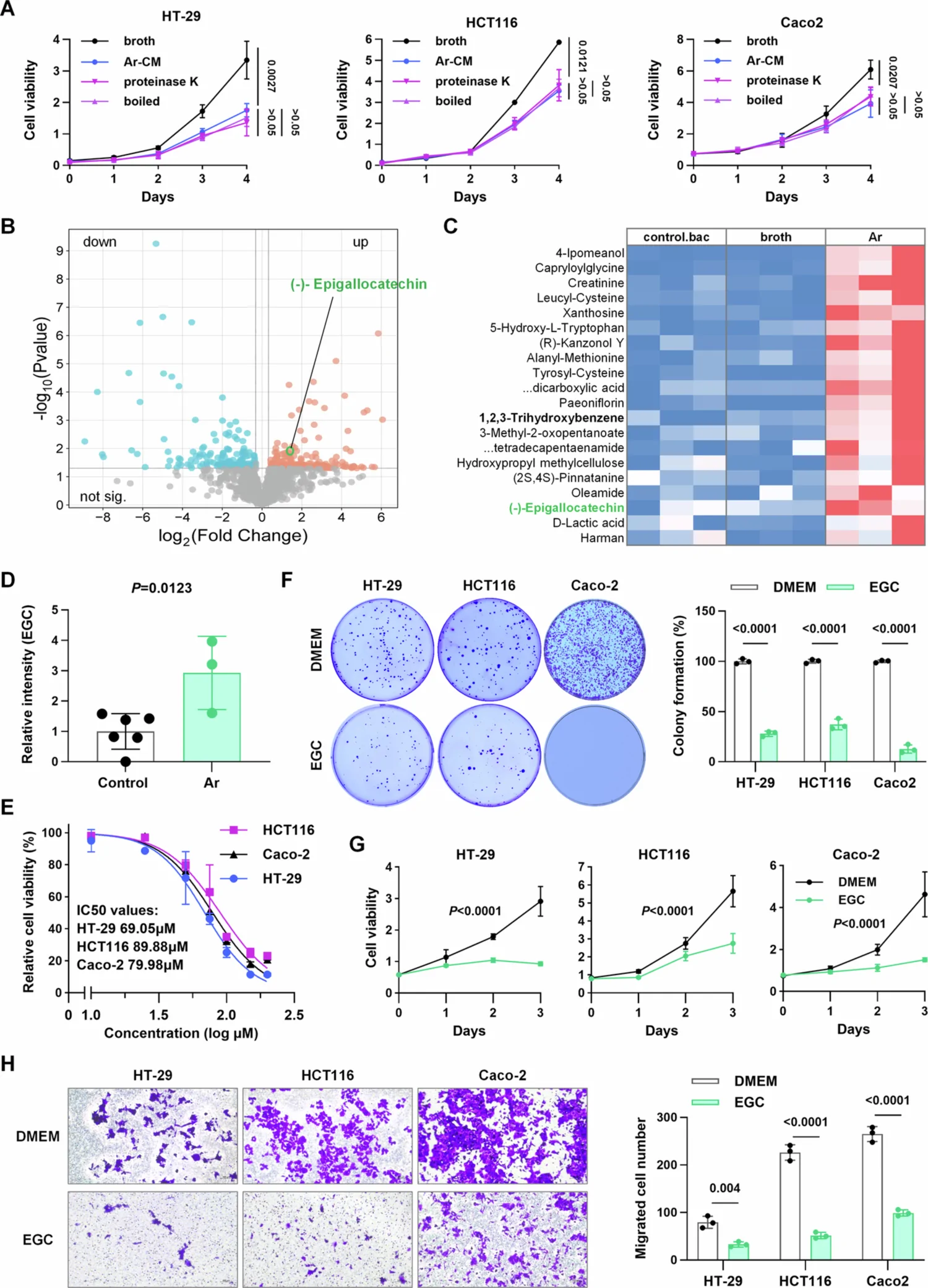
Epigallocatechin (EGC) is a potential functional metabolite of Ar. A. Boiling and proteinase K treatments showed no influence on the growth-inhibitory effect of Ar-conditioned medium (CM), indicating that the effective component is non-protein. B−D. Volcano plots and heatmap display differential metabolites identified in Ar-CM compared to broth and bacterial controls, among which EGC emerged as a significant candidate produced in Ar. E. Cell viability assays determined the half-maximal inhibitory concentration (IC_50_) of EGC in colon cancer cells. F−H. EGC significantly inhibited the colony formation ability (F), growth (G), and migration (H) of colon cancer cells.

Boiling or treatment with proteinase K did not alter the growth-inhibitory activity of the Ar conditioned medium (Figure 3A), indicating that the active component is non-proteinaceous. Untargeted LC–MS/MS identified 177 metabolites (Table S2) that were significantly increased in Ar compared to mock-cultured broth (Figure 3B). To identify metabolites that are relatively specifically produced by Ar, we further included another butyrate-producing bacterium (*F. prausnitzii*) as control and identified 20 metabolites significantly increased in Ar compared to broth and bacterial controls (Table S3). Among these, we are particularly interested in EGC, one of the major catechins found in green tea. EGC can be produced by certain flavonoid reductases that have been identified in some bacteria.^27^ Another metabolite, 1,2,3-Trihydroxybenzene, which is structurally related to EGC, is also specifically increased in Ar-conditioned medium (Figure 3C–D). Targeted LC–MS/MS analysis showed that EGC was undetectable in control medium but was present at approximately 300–400 ng/mL in Ar-conditioned medium (Figure S4A).

### EGC suppresses CRC cell growth *in vitro*

Catechins have demonstrated potential anti-cancer and cholesterol-modulating activities,^28^ ^,^ ^29^ but the specific functional role and mechanism of EGC remain uninvestigated. To investigate the effect of EGC, we performed an IC_50_ assay to evaluate its effective concentration on CRC cells, revealing IC_50_ values ranging from 70 to 90 μM on the tested cell lines (Figure 3E). Treatment with 80 μM EGC significantly inhibited the clonogenicity, growth, and migration of colon cancer cells (Figure 3F‒H). TUNEL staining showed that EGC treatment significantly increased apoptosis in CRC cells (Figure S4B). Western blot analysis further revealed that EGC reduced CCNE1, CCND1, and CDK6 expression, suggesting inhibition of G1-phase progression and G1/S transition (Figure S4C). These results demonstrate a tumor-suppressive role of EGC in colon cancer cells.

### EGC suppresses CRC development *in vivo* and modulates cholesterol metabolism in the gut

**Figure 4.**
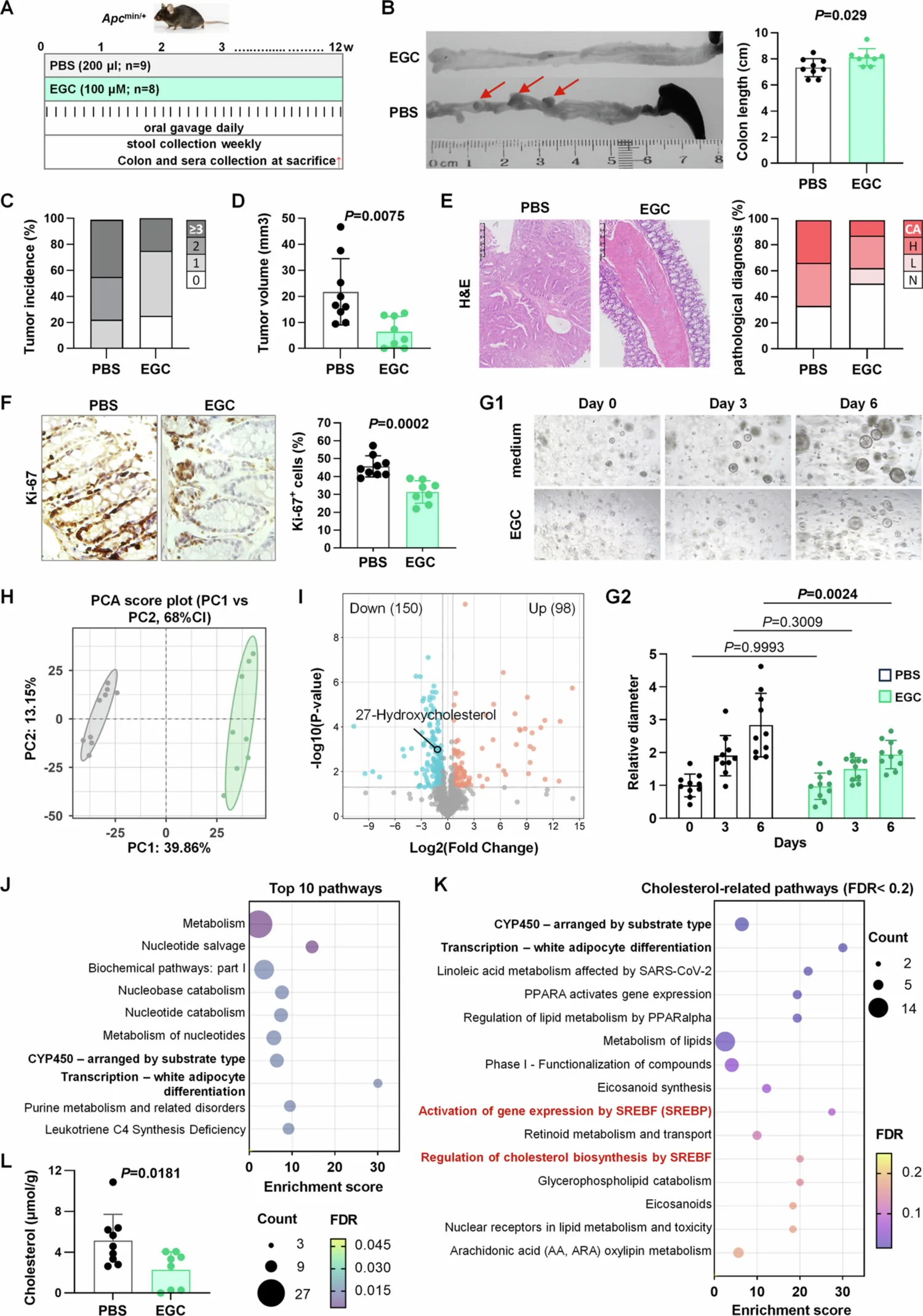
EGC exerts an inhibitory effect on CRC *in vivo*. A. Schematic diagram of the EGC treatment plan. B. Representative colon images and comparison of colon length between two treatment groups. C-D. EGC exerted an inhibitory effect on tumor incidence and tumor volume. E. Representative images of H&E staining of colon tissues and pathological diagnostics of the two treatment groups. CA, cancer; H, high-grade dysplasia; L, low-grade dysplasia; N, normal. F. Representative images of immunohistochemical Ki-67 staining of non-tumor colon tissues. Ki-67^+^​​​​​ cells were decreased in the EGC treatment group. G. Representative images of organoids treated with PBS or EGC. EGC significantly inhibit CRC organoids progression. H. Scatter plots of principal component analysis (PCA) showing distinct sample distributions between the EGC and PBS groups along the first two principal components. I. Volcano plot showing differential fecal metabolites between the EGC and control groups in mice. Hydroxycholesterol was significantly downregulated. J. Two of the top ten pathways (bold) enriched by differential metabolites between EGC and control groups are associated with cholesterol metabolism. K. All identified cholesterol-related pathways enriched by differential metabolites between EGC and control groups (FDR < 0.2). L. Cholesterol quantification assay indicates a significant decrease in fecal cholesterol levels in EGC-treated mice.

We further verified the function of EGC in a mouse model. EGC was administered to *Apc* ^min/+^ mice daily for 12 weeks (Figure 4A). Results showed that EGC increased colon length by 10.8% compared to control treatment (Figure 4B), similar to Ar administration. Importantly, EGC significantly reduced colon tumor incidence and tumor volume (Figure 4C–D) without any observed adverse effects. In addition, histological analysis revealed a lower incidence of high-grade dysplasia/cancer in EGC-treated mice compared to controls (Figure 4E). Ki-67 staining further demonstrated that EGC significantly reduced the proportion of actively proliferating cells in the non-tumor colon tissues harboring loss-of-function *Apc* mutations (Figure 4F). Furthermore, EGC treatment significantly decreased the growth of patient-derived CRC organoids (Figure 4G).

We further analyzed the fecal metabolomes using untargeted LC–MS/MS. PCA revealed distinct separation between EGC-treated and control groups (Figure 4H). We identified 248 differentially abundant metabolites by EGC treatment, including cholesterol derivatives such as 27-hydroxycholesterol (Figure 4I). Among the top 10 pathways enriched by these metabolites, two are associated with cholesterol/lipid metabolism (Figure 4J). Among the pathways with FDR < 0.2, at least 15 are related to cholesterol/lipid metabolism, two of which involved sterol regulatory element-binding protein (SREBP) signaling (Figure 4K and Table S4). Pathway-level mapping showed that EGC treatment altered cholesterol-derived metabolites, including 27-hydroxycholesterol, calcitriol, cortisol, and 6beta-hydroxytestosterone, as well as lipid-associated metabolites linked to fatty acid/PPAR–SREBP regulation (Figure S5). We further quantified fecal cholesterol levels and confirmed a significant reduction in EGC-treated mice compared to PBS controls (Figure 4L). These findings demonstrate that EGC recapitulates Ar's tumor-suppressive effects in CRC and also participates in the regulation of cholesterol homeostasis.

### Identification of downstream molecules regulated by EGC

**Figure 5.**
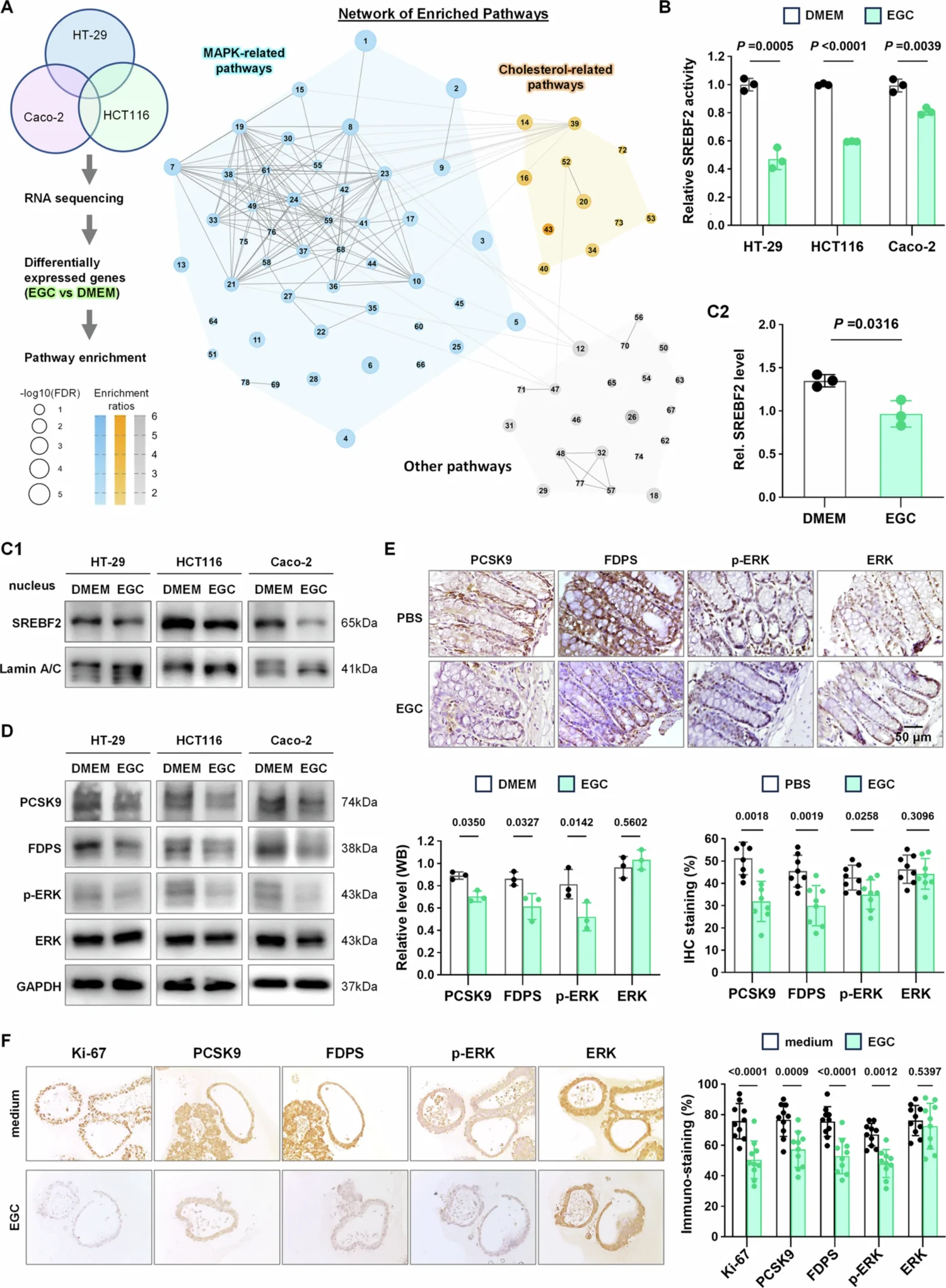
EGC modulates the SREBF2-mediated cholesterol metabolism and MAPK pathways. A. Pathways related to cholesterol metabolism and MAPK signaling were significantly enriched by EGC-induced differentially expressed genes. B. Transcriptional activity assays indicated that EGC treatment downregulated SREBF2 activity in colon cancer cells. C. EGC treatment reduced nuclear levels of SREBF2 in colon cancer cells. D. Western blot analysis demonstrated that EGC treatment downregulated protein levels of cholesterol metabolism-related genes (PCSK9 and FDPS) and the key MAPK signaling marker (p-ERK) in colon cancer cells. E. Immunohistochemical analysis showed that EGC treatment downregulated protein levels of PCSK9, FDPS, and p-ERK in mouse colon tissues. F. Immunohistochemical analysis showed that EGC treatment downregulated protein levels of Ki-67, PCSK9, FDPS, and p-ERK in patient-derived organoids.

We conducted transcriptome analysis to compare gene expression profiles between cells treated with and without EGC. Notably, pathway enrichment and network analysis revealed that genes dysregulated by EGC (log2|fold-change| > 0.15, FDR < 0.2) were predominantly enriched in cholesterol-related and MAPK-related pathways, including AMPK (AMP-activated protein kinase) signaling (Figure 5A and Table S5). Given that SREBP signaling is modulated by EGC, as indicated by fecal metabolomics (Figure 4K), and recognizing the close link between SREBF2 and AMPK signaling in CRC,^30^ we investigated whether EGC treatment regulates SREBF2 activity. SREBF2 transcriptional activity assays showed that EGC treatment significantly decreased SREBF2 activity across all three tested cell lines (Figure 5B). Correspondingly, nuclear SREBF2 levels were also significantly reduced by EGC treatment (Figure 5C). Supporting this, SREBF2 downstream genes were downregulated following EGC treatment according to RNA-seq data, including PCSK9 and FDPS, which are involved in cholesterol metabolism and linked to activated MAPK signaling.^31^ ^,^ ^32^ Further western blot analysis confirmed that, following EGC treatment, the protein levels of PCSK9, FDPS, and phosphorylated ERK (p-ERK; but not total ERK), were reduced in colon cancer cells (Figure 5D). IHC results also revealed that PCSK9, FDPS, and p-ERK levels were significantly reduced by EGC treatment in mouse colon tissues (Figure 5E). Moreover, Ki-67, PCSK9, FDPS, and p-ERK levels were significantly reduced by EGC treatment in patient-derived organoids (Figure 5F). These results demonstrate that EGC inhibits SREBF2 to modulate cholesterol metabolism and suppresses MAPK signaling.

### EGC binds to precursor SREBF2 to influence its activation

**Figure 6.**
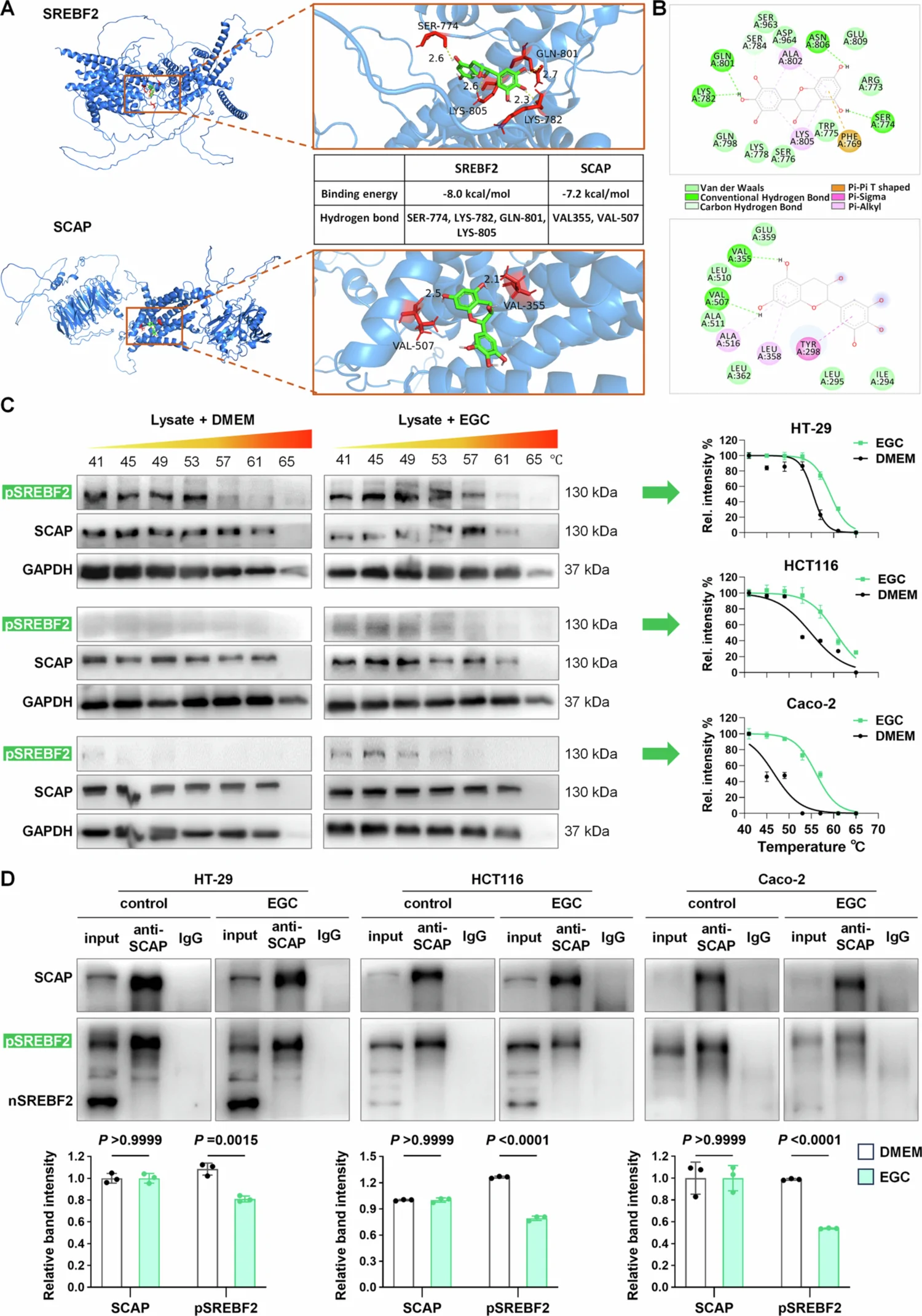
EGC binds to SREBF2 to impede its binding with SCAP. A. Molecular docking analysis predicted binding energies of −8.0 kcal/mol between EGC and SREBF2, and −7.2 kcal/mol between EGC and SCAP, with more hydrogen bonds identified between EGC and SREBF2. B. More additional chemical bonds were predicted between EGC and SREBF2 compared to those between EGC and SCAP. C. Cellular thermal shift assays demonstrated enhanced thermal stability of precursor SREBF2 (pSREBF2) in the presence of EGC, supporting a binding interaction. D. Co-immunoprecipitation analysis showed that EGC treatment reduced the interaction between pSREBF2 and SCAP.

To investigate how EGC regulates SREBF2 transcriptional activity, molecular docking analysis was conducted. SREBF2 is synthesized as a full-length precursor protein of approximately 125–130 kDa. After SCAP (SREBP cleavage-activating protein)-dependent trafficking and proteolytic cleavage, the mature transcriptionally active nuclear form of SREBF2 is generated and detected at approximately 60–68 kDa. Given that precursor SREBF2 (pSREBF2) and SCAP function as a complex to facilitate cleavage and activation of SREBF2, we conducted docking analyzes for both proteins. The binding energy of EGC to pSREBF2 is −8.0 kcal/mol, with SER-774, LYS-782, GLN-801, and LYS-805 engaged in hydrogen-bond interactions (Figure 6A). The binding energy of SCAP and EGC is −7.2  kcal/mol, with VAL355 and VAL-507 forming hydrogen bonds. There are also more predicted chemical bonds between pSREBF2 and EGC than between SCAP and EGC (Figure 6B). CETSA results indicated that under elevated temperatures, pSREBF2 exhibited greater stability in EGC-treated samples than in control treatments (Figure 6C), while no significant changes in the stability of SCAP or nuclear SREBF2 were observed (Figure S6A-B). We performed co-IP assays to further validate the regulatory effect of EGC on pSREBF2. Results showed that pSREBF2 bound to SCAP, as normalized to pulled-down SCAP levels, was significantly reduced in the presence of EGC, indicating reduced interaction between pSREBF2 and SCAP by EGC (Figure 6D). These results indicate that EGC binds to pSREBF2, enhancing its stability and impeding its interaction with SCAP, thereby resulting in reduced cleavage and activation of SREBF2.

### SREBF2 and its downstream genes mediate cholesterol metabolism modulation and MAPK suppression by EGC

**Figure 7.**
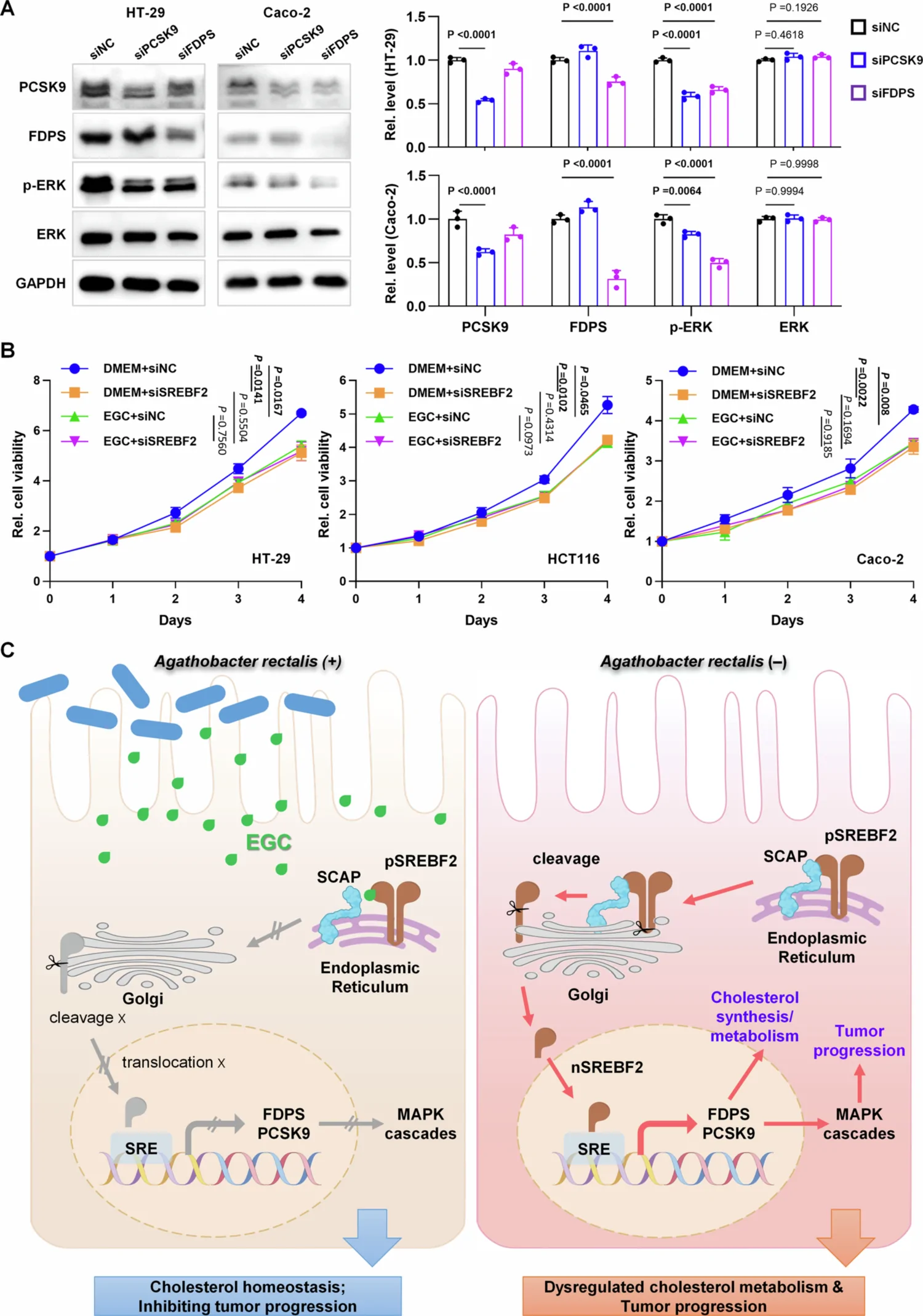
FDPS/PCSK9 knockdown suppresses MAPK activation and schematic model of the Ar–EGC–SREBF2 axis in CRC. A. Western blot analysis and quantification of PCSK9, FDPS, p-ERK, and ERK levels in HT-29 and Caco-2 cells. GAPDH was used as the loading control. B. SREBF2 knockdown reduced CRC cell viability and largely abolished the additional anti-proliferative effect of EGC. C. Schematic representation of how Ar suppresses CRC via modulation of cholesterol metabolism through EGC.

Although downstream SREBF2 signaling was inhibited, total and phosphorylated AMPK levels remained unchanged (Figure S7A), indicating that EGC does not trigger an AMPK-mediated metabolic stress response. Consistent with the effects of EGC, Ar-conditioned medium significantly reduced nuclear SREBF2 levels in HCT116 and Caco-2 cells (Figure S7B) and decreased PCSK9, FDPS, and p-ERK protein levels without affecting total ERK (Figure S7C), indicating that Ar-conditioned medium also suppresses the SREBF2-mediated cholesterol metabolism and MAPK signaling axis. To determine the functional relevance of FDPS and PCSK9 downregulation, we knocked down FDPS or PCSK9 in CRC cells using siRNAs. Knockdown of either gene significantly suppressed CRC cell proliferation and migration relative to control (Figure S8A-B). Western blot analysis further showed that FDPS or PCSK9 knockdown reduced MAPK pathway activation, as indicated by decreased p-ERK levels (Figure 7A). These findings suggest that EGC and Ar-conditioned medium act through downregulation of FDPS and PCSK9 to suppress MAPK pathway activation in CRC.

To determine whether SREBF2 is functionally required for the antitumor activity of EGC, we knocked down SREBF2 expression in CRC cells and examined whether SREBF2 depletion could phenocopy EGC treatment. In all tested cells, SREBF2 knockdown significantly reduced cell viability (Figure 7B) and migration (Figure S9) compared with the control group, with inhibitory effects comparable to those of EGC treatment. Combined SREBF2 knockdown and EGC treatment did not further suppress cell viability or migration compared with either treatment alone, indicating that SREBF2 depletion functionally mimics EGC treatment and that the antitumor effects of EGC are largely dependent on SREBF2.

## Discussion

In this study, we elucidate a previously underappreciated antitumor role of the commensal Ar in CRC and delineate its underlying mechanisms (Figure 7C). Importantly, unlike most previous studies that primarily attribute the beneficial effects of Ar to butyrate, we identified a novel metabolite, EGC, which plays a key role in mediating Ar's tumor-suppressive effects against CRC by modulating cholesterol metabolism and inhibiting MAPK signaling.

Ar is a well-recognized butyrate-producing bacterium. Our previous research has demonstrated that its production of butyrate also exerts antitumor effects by inhibiting cholesterol synthesis in intestinal cells.^30^ In the present study, by comparing Ar with another butyrate-producing bacterium, we revealed to our knowledge that Ar promotes EGC accumulation, which mediates its unique tumor-suppressive activity. This novel mechanism, in which Ar exerts protective effects on the host through beneficial metabolites beyond butyrate, fills a gap in our understanding of gut bacterial functionality. In addition to EGC, Ar, as well as other butyrate-producing bacteria, may produce other beneficial metabolites beyond butyrate critical for host health, which warrants future investigation.

Using untargeted LC–MS/MS coupled with subsequent functional and mechanistic studies, we identified EGC as a key metabolite responsible for Ar's anti-cancer effects. EGC is a catechin present in green tea, though its abundance is considerably lower than that of (–)-epigallocatechin gallate (EGCG).^33^ Moreover, the brewing temperature influences the release of different catechins.^34^ As a result, EGCG is the primary catechin obtained from green tea consumption. Current catechin research has largely focused on EGCG, which has been reported to possess various physiological functions, including antioxidant, cholesterol-lowering, hypoglycemic, and hypotensive activities, along with certain anti-cancer properties.^28^ ^,^ ^29^ ^,^ ^35^ ^,^ ^36^ However, absorption of intact EGCG in the body has been reported to be very low.^37^ ^,^ ^38^ As a catechin lacking the galloyl moiety present in EGCG, EGC may be more readily absorbed and could potentially mediate anti-cancer effects more efficiently. Moreover, although green tea consumption may provide a certain amount of EGC, the continuous, localized, and endogenous production of EGC by gut-colonized Ar may offer a distinct advantage over dietary intake alone.

Using targeted LC–MS/MS, we further confirmed that EGC was detectable in Ar-conditioned medium but absent in control medium. As EGC may be susceptible to degradation outside optimal conditions, a higher concentration (80 µM) was used for *in vitro* assays based on the IC_50_ results. Although the physiological concentration of EGC in the gut is likely lower than that used *in vitro*, Ar-derived EGC may exert antitumor effects *in vivo* through sustained low-dose exposure, potential local accumulation, and synergistic interactions with other Ar-derived metabolites. While the mechanisms underlying EGCG's anti-cancer or cholesterol-modulating actions remain incompletely understood, the specific functional mechanisms of EGC had been even less defined. Our study demonstrates the antitumor activity of EGC and supports a mechanism in which EGC interferes with the SCAP–SREBF2 axis, thereby inhibiting SREBF2 proteolytic activation, nuclear translocation, and transcriptional activity. Molecular docking suggested a potential interaction between EGC and pSREBF2 as well as SCAP. Further CETSA and Co-IP assays showed that EGC increased the thermal stability of pSREBF2, but not SCAP, and weakened its interaction with SCAP. However, the current data do not directly determine the binding affinity or kinetics between EGC and pSREBF2, and further biophysical studies are needed to clarify their direct binding properties. The role of SREBF2 in cholesterol regulation and its tumor-promoting effects in CRC and other cancers are well-established.^39^ ^-^ ^42^ Furthermore, we confirmed that EGC downregulates the expression of two SREBF2 downstream genes, FDPS and PCSK9, and suppresses MAPK pathway activation. These genes have been reported to participate in cholesterol synthesis and transport, activate MAPK signaling, and thereby promote cancer progression.^31^ ^,^ ^32^ ^,^ ^43-45^

Recent studies have increasingly recognized cholesterol metabolism as an important metabolic vulnerability in CRC. SREBF2 is a central transcriptional regulator of cholesterol biosynthesis and mevalonate metabolism, which can support tumor cell proliferation by maintaining membrane biogenesis, lipid metabolic remodeling, and oncogenic signaling.^46^ Consistently, previous studies have shown that PCSK9 promotes APC/KRAS-mutant CRC through the GGPP–KRAS–MEK/ERK signaling axis, whereas PCSK9 depletion or inhibition suppresses MEK/ERK activation.^47^ These findings support a functional link between cholesterol/mevalonate metabolic regulation and oncogenic MAPK signaling in CRC. For FDPS, increased enzymatic activity has been reported in human CRC tissues, and FDPS inhibition suppresses colon cancer cell growth and promotes apoptosis.^48^ In addition, studies in other tumor or proliferative cell models have shown that FDPS can regulate ERK/MAPK-related signaling through small GTPase-dependent mechanisms.^49^ Based on these published findings and our new experimental data, we propose a refined model in which EGC inhibits SREBF2-dependent cholesterol/mevalonate metabolic signaling, downregulates FDPS and PCSK9, and consequently attenuates MAPK pathway activation, thereby contributing to reduced CRC cell growth and migration. In parallel, recent reviews have emphasized that the gut microbiota regulates host cellular metabolism in digestive diseases through diverse microbial metabolites and metabolic pathways, including lipid-related pathways.^50^ Compared with these studies, our work provides direct experimental evidence linking a defined commensal bacterium, Ar, to a specific bioactive metabolite, EGC, and further identifies pSREBF2 as a molecular target of EGC. Our study therefore extends the current understanding of microbiota-regulated cholesterol metabolism by demonstrating a gut microbe–metabolite–SREBF2 axis that suppresses CRC progression.

Whether reduced Ar abundance in CRC patients is a causal contributor to tumorigenesis or a consequence of disease-associated dysbiosis remains to be determined. On one hand, Ar depletion may weaken microbiota-mediated protection by reducing beneficial metabolites such as butyrate and EGC, thereby facilitating dysregulated cholesterol metabolism and proliferative signaling. On the other hand, tumor-associated inflammation, altered nutrient availability, and disruption of the intestinal microenvironment may secondarily reduce Ar abundance. Thus, the decreased fecal Ar abundance observed in CRC patients should be interpreted as an association rather than direct evidence of causality. Nevertheless, our animal supplementation and mechanistic investigation provide functional evidence supporting a protective role of Ar against CRC.

Our findings advance the concept that specific gut commensals can supply bioactive metabolites and rebalance host lipid metabolism and proliferative signaling. Clinically, the observed reduction of fecal Ar in CRC patients and the tumor-suppressive activity of Ar/EGC in preclinical models suggest that Ar abundance or EGC levels may have potential biomarker value. These findings may also provide a basis for the future development of Ar- or EGC-based strategies as adjunctive approaches for CRC prevention or treatment. However, because the current evidence is mainly derived from association analyzes and preclinical models, further studies in large longitudinal clinical cohorts and well-designed translational models are required to validate the causal relationship, clinical relevance, safety, delivery feasibility, and therapeutic efficacy of Ar/EGC-based interventions.

In summary, we have revealed a gut microbe–metabolite axis in which Ar and its metabolite (–)-epigallocatechin suppress CRC progression by antagonizing SREBF2 activation, thereby modulating cholesterol metabolism and inhibiting MAPK signaling. These findings provide mechanistic insights into microbiome-mediated tumor control and may provide a basis for future development of Ar/EGC-based interventions targeting cholesterol metabolism in CRC management.

## Supplementary Material

Supplementary Table4.xlsx Supplementary Table4.xlsx

Supplementery Table 3.xlsx Supplementery Table 3.xlsx

Supplementary Table1.xlsx Supplementary Table1.xlsx

Supplementary Figures.pdf Supplementary Figures.pdf

SUPPLEMENTARY METHODS.pdf SUPPLEMENTARY METHODS.pdf

Supplementery Table 2.xlsx Supplementery Table 2.xlsx

Supplementary Table5.xlsx Supplementary Table5.xlsx

ARRIVE Author Checklist.pdf ARRIVE Author Checklist.pdf

## Data Availability

Untargeted metabolomic data of bacterial culture supernatants and mock-cultured medium controls have been deposited in the China National GeneBank DataBase (CNGBdb) Sequence Archive (CNSA) (https://db.cngb.org/cnsa/) under accession CNP0007790. Untargeted metabolomic data of stool samples from mice have been deposited in the National Genomics Data Center (https://ngdc.cncb.ac.cn/) under accession code PRJCA057060. RNA-sequencing data have been deposited in the NCBI Sequence Read Archive (SRA) database (https://www.ncbi.nlm.nih.gov/sra) with the accession numbers PRJNA1465052.

## References

[1] Collaborators GBDCC Global, regional, and national burden of colorectal cancer and its risk factors, 1990-2019: a systematic analysis for the global burden of disease study 2019. Lancet Gastroenterol Hepatol. 2022;7(7):627–647. doi: 10.1016/S2468-1253(22)00044-9. 35397795 PMC9192760

[2] Li Q , Geng S , Luo H , Wang W , Mo Y , Song G , Sheng J , Xu B . Signaling pathways involved in colorectal cancer: pathogenesis and targeted therapy. Signal Transduct Target Ther. 2024;9(1):266. doi: 10.1038/s41392-024-01953-7. 39370455 PMC11456611

[3] Li XP , Qu J , Teng XQ , Zhuang H , Dai Y , Yang Z . The emerging role of super-enhancers as therapeutic targets in the digestive system tumors. Int J Biol Sci. 2023;19(4):1036–1048. doi: 10.7150/ijbs.78535. 36923930 PMC10008685

[4] Tong G , Peng T , Chen Y , Sha L , Dai H , Xiang Y , Zou Z , He H , Wang S . Effects of GLP-1 receptor agonists on biological behavior of colorectal cancer cells by regulating PI3K/AKT/mTOR signaling pathway. Front Pharmacol. 2022;13:901559. doi: 10.3389/fphar.2022.901559. 36034798 PMC9399678

[5] Mzoughi S , Schwarz M , Wang X , Demircioglu D , Ulukaya G , Mohammed K , Zorgati H , Torre D , Tomalin LE , Di Tullio F , et al. Oncofetal reprogramming drives phenotypic plasticity in WNT-dependent colorectal cancer. Nat Genet. 2025;57(2):402–412. doi: 10.1038/s41588-024-02058-1. 39930084 PMC11821538

[6] White MT , Sears CL . The microbial landscape of colorectal cancer. Nat Rev Microbiol. 2024;22(4):240–254. doi: 10.1038/s41579-023-00973-4. 37794172

[7] Cheng Y , Ling Z , Li L . The intestinal microbiota and colorectal cancer. Front Immunol. 2020;11:615056. doi: 10.3389/fimmu.2020.615056. 33329610 PMC7734048

[8] Brennan CA , Garrett WS . Gut microbiota, inflammation, and colorectal cancer. Annu Rev Microbiol. 2016;70:395–411. doi: 10.1146/annurev-micro-102215-095513. 27607555 PMC5541233

[9] Louis P , Hold GL , Flint HJ . The gut microbiota, bacterial metabolites and colorectal cancer. Nat Rev Microbiol. 2014;12(10):661–672. doi: 10.1038/nrmicro3344. 25198138

[10] Wang J , Zhu N , Su X , Gao Y , Yang R . Gut-microbiota-derived metabolites maintain gut and systemic immune homeostasis. Cells. 2023;12(5):793. doi: 10.3390/cells12050793. 36899929 PMC10000530

[11] Gosai V , Ambalam P , Raman M , Kothari C , Vyas B , Sheth NR . Protective effect of lactobacillus rhamnosus 231 against N-Methyl-N'-nitro-N-nitrosoguanidine in animal model. Gut Microbes. 2011;2(6):319–325. doi: 10.4161/gmic.18755. 22157237

[12] Zhang M , Fan X , Fang B , Zhu C , Zhu J , Ren F . Effects of lactobacillus salivarius ren on cancer prevention and intestinal microbiota in 1, 2-dimethylhydrazine-induced rat model. J Microbiol. 2015;53(6):398–405. doi: 10.1007/s12275-015-5046-z. 26025172

[13] Zuo G , Hao B . Whole-genome-based phylogeny supports the objections against the reclassification of eubacterium rectale to agathobacter rectalis. Int J Syst Evol Microbiol. 2016;66(6):2451–2451. doi: 10.1099/ijsem.0.001047. 27032096

[14] Lu H , Xu X , Fu D , Gu Y , Fan R , Yi H , He X , Wang C , Ouyang B , Zhao P , et al. Butyrate-producing eubacterium rectale suppresses lymphomagenesis by alleviating the TNF-induced TLR4/MyD88/NF-κB axis. Cell Host Microbe. 2022;30(8):1139–1150.e1137. doi: 10.1016/j.chom.2022.07.003. 35952646

[15] Yeoh YK , Zuo T , Lui GC , Zhang F , Liu Q , Li AY , Chung AC , Cheung CP , Tso EY , Fung KS , et al. Gut microbiota composition reflects disease severity and dysfunctional immune responses in patients with COVID-19. Gut. 2021;70(4):698–706. doi: 10.1136/gutjnl-2020-323020. 33431578 PMC7804842

[16] Liu Y , Chan MTV , Chan FKL , Wu WKK , Ng SC , Zhang L . Lower gut abundance of eubacterium rectale is linked to COVID-19 mortality. Front Cell Infect Microbiol. 2023;13, 10.3389/fcimb.2023.1249069. PMC10512258 37743871

[17] Islam SMS , Ryu HM , Sayeed HM , Byun H , Jung J , Kim H , Suh C , Sohn S . Eubacterium rectale attenuates HSV-1 induced systemic inflammation in mice by inhibiting CD83. Front Immunol. 2021;12:712312. doi: 10.3389/fimmu.2021.712312. 34531862 PMC8438521

[18] Liu N , Chen L , Yan M , Tao Q , Wu J , Zhang W , Peng C . Eubacterium rectale improves the efficacy of Anti-PD1 immunotherapy in melanoma via l-Serine-Mediated NK cell activation. Research (Wash D C). 2023;6:0127. doi: 10.34133/research.0127. 37223471 PMC10202379

[19] Duncan SH , Flint HJ . Proposal of a neotype strain (A1-86) for eubacterium rectale. Request for an opinion. Int J Syst Evol Microbiol. 2008;58(Pt 7):1735–1736. doi: 10.1099/ijs.0.2008/004580-0. 18599726

[20] Tsoi H , Chu ESH , Zhang X , Sheng J , Nakatsu G , Ng SC , Chan AW , Sung JJ , Yu J . Peptostreptococcus anaerobius induces intracellular cholesterol biosynthesis in colon cells to induce proliferation and causes dysplasia in mice. Gastroenterology. 2017;152(6):1419–1433 e1415. doi: 10.1053/j.gastro.2017.01.009. 28126350

[21] Zeller G , Tap J , Voigt AY , Sunagawa S , Kultima JR , Costea PI , Amiot A , Böhm J , Brunetti F , Habermann N , et al. Potential of fecal microbiota for early-stage detection of colorectal cancer. Mol Syst Biol. 2014;10(11):766. doi: 10.15252/msb.20145645. 25432777 PMC4299606

[22] Gupta A , Dhakan DB , Maji A , Saxena R , P.k. VP , Mahajan S , Pulikkan J , Kurian J , Gomez AM , Scaria J , et al. Association of flavonifractor plautii, a flavonoid-degrading bacterium, with the gut microbiome of colorectal cancer patients in India. mSystems. 2019;4(6). doi: 10.1128/msystems.00438-19. PMC7407896 31719139

[23] Beghini F , McIver LJ , Blanco-Miguez A , Blanco-Míguez A , Dubois L , Asnicar F , Maharjan S , Mailyan A , Manghi P , Scholz M , et al. Integrating taxonomic, functional, and strain-level profiling of diverse microbial communities with bioBakery 3. eLife. 2021;10. doi: 10.7554/eLife.65088. PMC8096432 33944776

[24] Duncan SH , Hold GL , Harmsen HJM , Stewart CS , Flint HJ . Growth requirements and fermentation products of fusobacterium prausnitzii, and a proposal to reclassify it as faecalibacterium prausnitzii gen. Nov., comb. Nov. Int J Syst Evol Microbiol. 2002;52(Pt 6):2141–2146. doi: 10.1099/00207713-52-6-2141. 12508881

[25] Pang Z , Lu Y , Zhou G , Hui F , Xu L , Viau C , Spigelman AF , MacDonald PE , Wishart DS , Li S , et al. MetaboAnalyst 6.0: towards a unified platform for metabolomics data processing, analysis and interpretation. Nucleic Acids Res. 2024;52(W1):W398–W406. doi: 10.1093/nar/gkae253. 38587201 PMC11223798

[26] Che N , Li M , Liu X , Cui CA , Gong J , Xuan Y . Macelignan prevents colorectal cancer metastasis by inhibiting M2 macrophage polarization. Phytomedicine. 2024;122:155144. doi: 10.1016/j.phymed.2023.155144. 37925889

[27] Goris T , Cuadrat RRC , Braune A . Flavonoid-modifying capabilities of the human gut microbiome-an in silico study. Nutrients. 2021;13(8):2688. doi: 10.3390/nu13082688. 34444848 PMC8398226

[28] Shirakami Y , Shimizu M . Possible mechanisms of Green tea and its constituents against cancer. Molecules. 2018;23(9):2284. doi: 10.3390/molecules23092284. 30205425 PMC6225266

[29] Bursill CA , Roach PD . Modulation of cholesterol metabolism by the Green tea polyphenol (-)-epigallocatechin gallate in cultured human liver (HepG2) cells. J Agric Food Chem. 2006;54(5):1621–1626. doi: 10.1021/jf051736o. 16506810

[30] Sun Y , Lu J , Tung Lau EY , Zeng Y , Lam Li SW , Au TH , Ye S , Zhou T , Chan FK , Liang JQ . Fusobacterium nucleatum enhances cholesterol biosynthesis in colorectal cancer via miR-130a-3p-mediated AMPK inhibition, a process counteracted by butyrate. Cancer Lett. 2025;627:217810. doi: 10.1016/j.canlet.2025.217810. 40414519

[31] Xu B , Li S , Fang Y , Zou Y , Song D , Zhang S , Cai Y . Proprotein convertase Subtilisin/Kexin type 9 promotes gastric cancer metastasis and suppresses apoptosis by facilitating MAPK signaling pathway through HSP70 up-regulation. Front Oncol. 2020;10:609663. doi: 10.3389/fonc.2020.609663. 33489919 PMC7817950

[32] Guo X , Cao Y , He Q , Chen L , Wang Q , Zhang J , Lv W , Zhou X . Modulation of the RAC1/MAPK/ERK signalling pathway by farnesyl diphosphate synthase regulates granulosa cells proliferation in polycystic ovary syndrome. Hum Cell. 2024;37(3):689–703. doi: 10.1007/s13577-024-01050-5. 38551774

[33] Nain CW , Mignolet E , Herent MF , Quetin-Leclercq J , Debier C , Page MM , Larondelle Y . The catechins profile of Green tea extracts affects the antioxidant activity and degradation of catechins in DHA-Rich oil. Antioxidants (Basel). 2022;11(9):1844. doi: 10.3390/antiox11091844. 36139917 PMC9495874

[34] Bazinet L , Labbé D , Tremblay A . Production of Green tea EGC- and EGCG-enriched fractions by a two-step extraction procedure. Sep Purif Technol. 2007;56(1):53–56. doi: 10.1016/j.seppur.2007.01.014.

[35] Ma Y , Ding SJ , Fei YQ , Liu G , Jang HM , Fang J . Antimicrobial activity of anthocyanins and catechins against foodborne pathogens escherichia coli and salmonella. Food Control. 2019;106:106712. doi: 10.1016/j.foodcont.2019.106712.

[36] Gan RY , Li HB , Sui ZQ , Corke H . Absorption, metabolism, anti-cancer effect and molecular targets of epigallocatechin gallate (EGCG): an updated review. Crit Rev Food Sci Nutr. 2018;58(6):924–941. doi: 10.1080/10408398.2016.1231168. 27645804

[37] Kohri T , Matsumoto N , Yamakawa M , Suzuki M , Nanjo F , Hara Y , Oku N . Metabolic fate of (-)-[4-(3)H]epigallocatechin gallate in rats after oral administration. J Agric Food Chem. 2001;49(8):4102–4112. doi: 10.1021/jf001491+. 11513717

[38] Li C , Lee MJ , Sheng S , Meng X , Prabhu S , Winnik B , Huang B , Chung JY , Yan S , Ho C , et al. Structural identification of two metabolites of catechins and their kinetics in human urine and blood after tea ingestion. Chem Res Toxicol. 2000;13(3):177–184. doi: 10.1021/tx9901837. 10725114

[39] Mok EHK , Leung CON , Zhou L , Lei MML , Tong M , Wong TL , Lau EYT , Ng IOL , Ding J , Yun JP , et al. Caspase-3-Induced activation of SREBP2 drives drug resistance via promotion of cholesterol biosynthesis in hepatocellular carcinoma. Cancer Res. 2022;82(17):3102–3115. doi: 10.1158/0008-5472.CAN-21-2934. 35767704

[40] Wang X , Lee D , Xu H , Sui Y , Meisenhelder J , Hunter T . PIN1 prolyl isomerase promotes initiation and progression of bladder cancer through the SREBP2-Mediated cholesterol biosynthesis pathway. Cancer Discov. 2025;15(3):633–655. doi: 10.1158/2159-8290.CD-24-0866. 39808064 PMC11875963

[41] Wang R , Gilbert C , Tahiri H , Yang C , Landreville S , Hardy P . MiR-181a-driven downregulation of cholesterol biosynthesis through SREBP2 inhibition suppresses uveal melanoma metastasis. J Exp Clin Cancer Res. 2025;44(1):215. doi: 10.1186/s13046-025-03459-8. 40684202 PMC12275384

[42] Wen YA , Xiong X , Zaytseva YY , Napier DL , Vallee E , Li AT , Wang C , Weiss HL , Evers BM , Gao T . Downregulation of SREBP inhibits tumor growth and initiation by altering cellular metabolism in colon cancer. Cell Death Dis. 2018;9(3):265. doi: 10.1038/s41419-018-0330-6. 29449559 PMC5833501

[43] Alannan M , Seidah NG , Merched AJ . PCSK9 in liver cancers at the crossroads between lipid metabolism and immunity. Cells. 2022;11(24):4132. doi: 10.3390/cells11244132. 36552895 PMC9777286

[44] Silvente-Poirot S , Poirot M . Cholesterol epoxide hydrolase and cancer. Curr Opin Pharmacol. 2012;12(6):696–703. doi: 10.1016/j.coph.2012.07.007. 22917620

[45] Sun D , Wang C , Long S , Ma Y , Guo Y , Huang Z , Chen X , Zhang C . C/EBP-beta-activated microRNA-223 promotes tumour growth through targeting RASA1 in human colorectal cancer. Br J Cancer. 2015;112(9):1491–1500. doi: 10.1038/bjc.2015.107. 25867276 PMC4453668

[46] Xue L , Qi H , Zhang H , Ding L , Huang Q , Zhao D , Wu BJ , Li X . Targeting SREBP-2-Regulated mevalonate metabolism for cancer therapy. Front Oncol. 2020;10:1510. doi: 10.3389/fonc.2020.01510. 32974183 PMC7472741

[47] Wong CC , Wu JL , Ji F , Kang W , Bian X , Chen H , Chan L , Luk STY , Tong S , Xu J , et al. The cholesterol uptake regulator PCSK9 promotes and is a therapeutic target in APC/KRAS-mutant colorectal cancer. Nat Commun. 2022;13(1):3971. doi: 10.1038/s41467-022-31663-z. 35803966 PMC9270407

[48] Notarnicola M , Messa C , Cavallini A , Bifulco M , Tecce MF , Eletto D , Di Leo A , Montemurro S , Laezza C , Caruso MG . Higher farnesyl diphosphate synthase activity in human colorectal cancer inhibition of cellular apoptosis. Oncology. 2004;67(5-6):351–358. doi: 10.1159/000082918. 15713990

[49] Seshacharyulu P , Rachagani S , Muniyan S , Siddiqui JA , Cruz E , Sharma S , Krishnan R , Killips BJ , Sheinin Y , Lele SM , et al. FDPS cooperates with PTEN loss to promote prostate cancer progression through modulation of small GTPases/AKT axis. Oncogene. 2019;38(26):5265–5280. doi: 10.1038/s41388-019-0791-9. 30914801 PMC6597298

[50] Zhan XH , Cao ZY , Wang SH , Xia H , Wu H , He Z , Fang X . Role of gut microbiota in regulating host cell metabolism in digestive system diseases. TransMed. 2026;1:100019. doi: 10.1016/j.tmed.2026.100019.

