# Supplementary material for: Agathobacter rectalis suppresses colorectal tumorigenesis via an epigallocatechin–SREBF2 axis controlling cholesterol metabolism
Source: Gut Microbes. 2026 Aug 28;18(1):2724227. doi: 10.1080/19490976.2026.2724227 (PMC13531980; doi:10.1080/19490976.2026.2724227)
Supplement: Supplementary file 1 [file KGMI_A_2724227_SM1542.pdf]

Figure S1

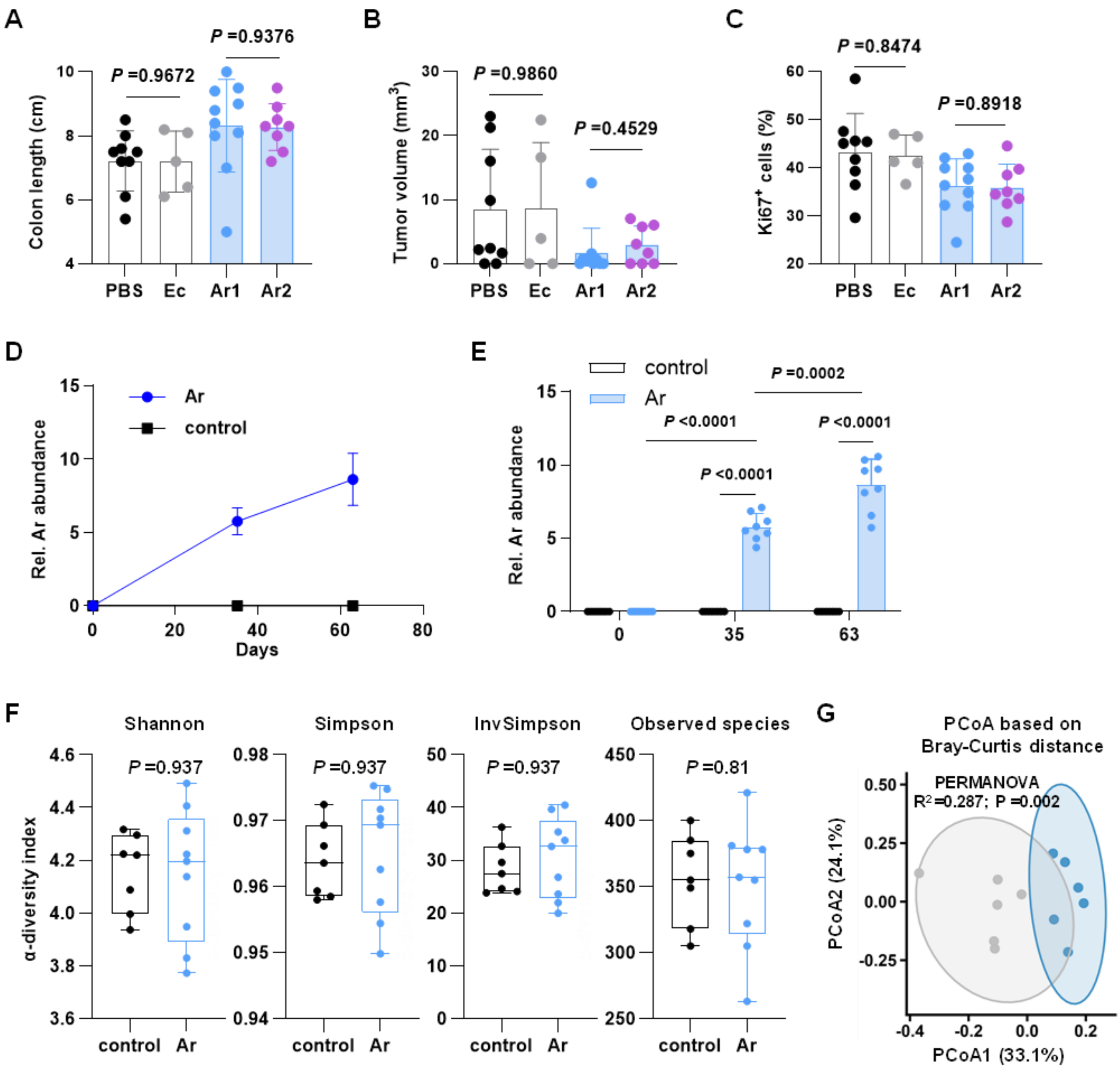

Supplementary Figure 1.

A-C. No significant differences were observed in colon length, tumor volume, or proportions of Ki67(+) cells between PBS and *E. coli* groups, or between our isolated Ar strain and Ar strain A1-86.

D-E. Species-specific qPCR was performed to quantify A. rectalis abundance in fecal samples collected from $Apc^{Min/+}$ mice. Each dot represents one mouse. Statistical significance was determined by unpaired two-tailed Student's t-test for two-group comparisons and one-way ANOVA with multiple-comparison correction for comparisons among multiple time points.

F. Alpha-diversity was assessed using the Shannon index, Simpson index, inverse Simpson index, and observed species richness based on species-level metagenomic profiles. Each point represents one mouse. Statistical significance was determined using the Wilcoxon rank-sum test. No significant differences were observed between the control and Ar groups.

G. Ar treatment significantly alters gut microbial community structure. Principal coordinate analysis (PCoA) based on Bray–Curtis distances showing differences in species-level fecal microbiome composition between the control and Ar groups. Each point represents one mouse. The separation between groups was assessed by PERMANOVA ($R^2 = 0.287$, $P = 0.002$).

Figure S2

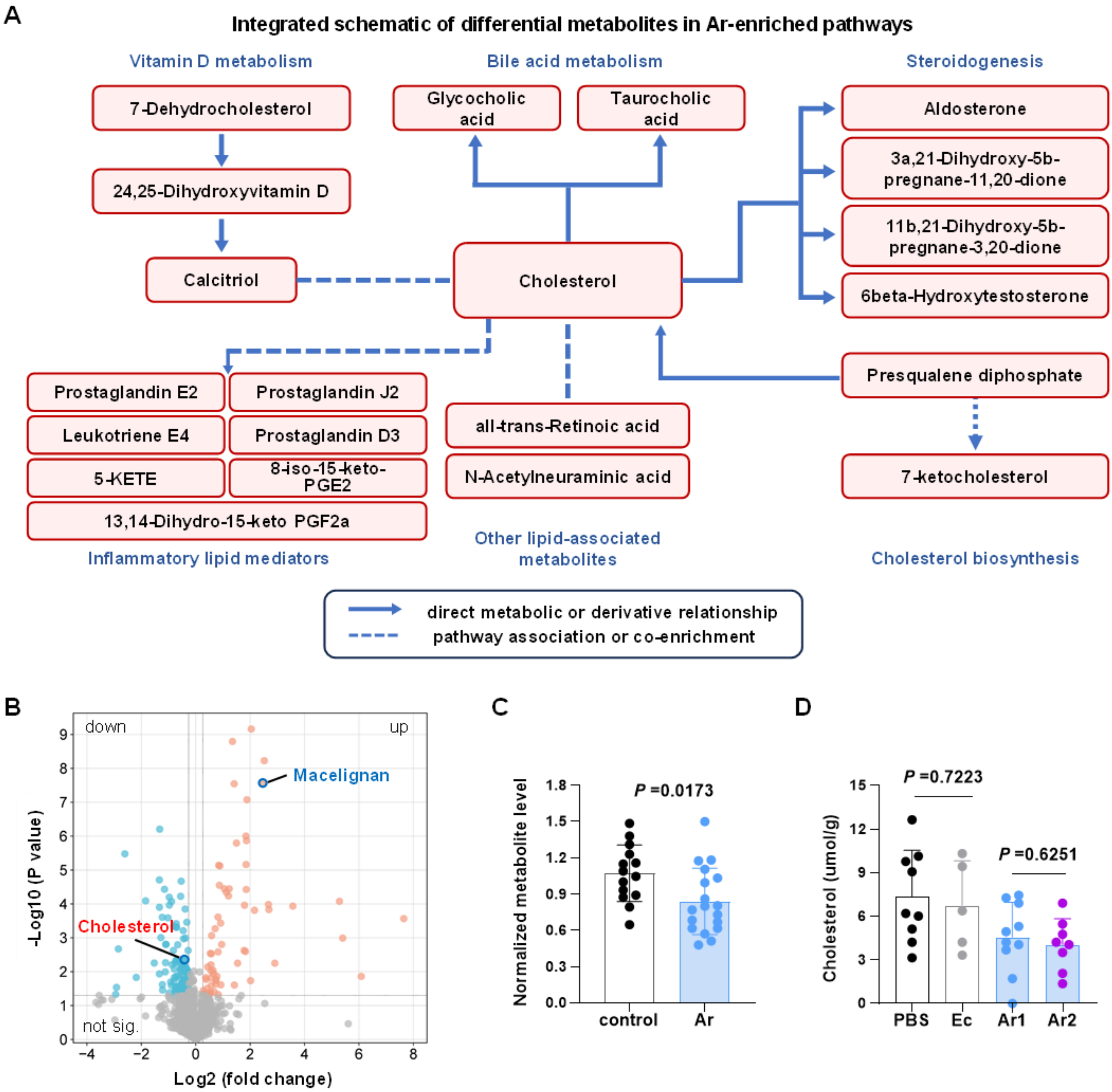

Supplementary Figure 2.

A. Differential metabolites were mapped to cholesterol- and lipid-related pathways. Red boxes indicate Ar-associated differential metabolites; solid arrows show direct metabolic relationships, and dashed lines indicate pathway association or co-enrichment.

B. Volcano plot showing differentially abundant fecal metabolites between Ar treatment and control groups in mice. Cholesterol was significantly downregulated, while the representative beneficial metabolite (macelignan) was upregulated.

C. Cholesterol levels were downregulated in Ar-treated mice compared to control-treated mice.

D. No significant differences were observed in cholesterol levels between PBS and E. coli groups, or between our isolated Ar strain and Ar strain A1-86.

Figure S3

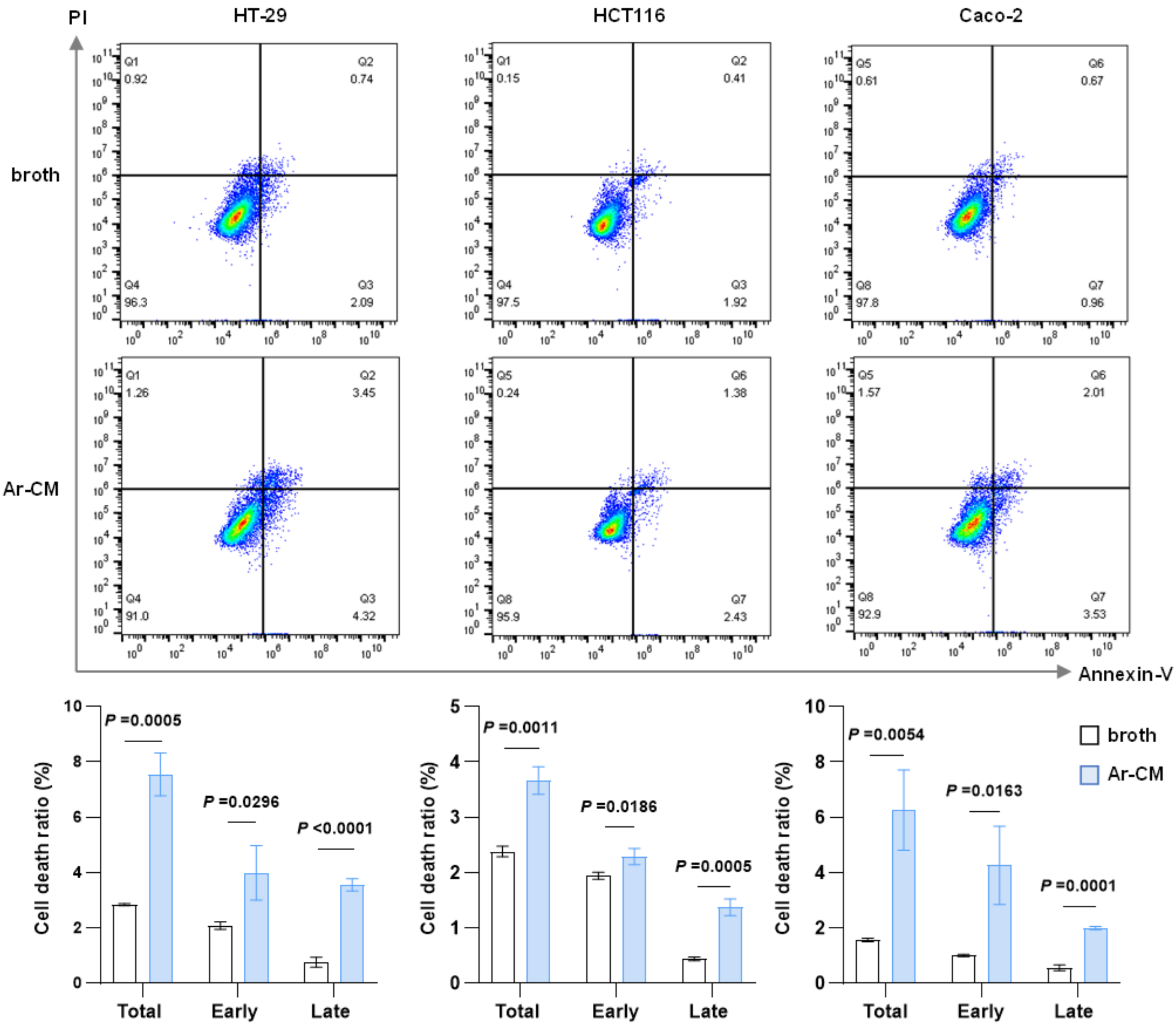

Supplementary Figure 3.

CRC cells were treated with control broth or Ar-CM, and apoptosis was measured by Annexin V/PI flow cytometry. Representative plots and quantification of total, early, and late apoptotic cells are shown.

Figure S4

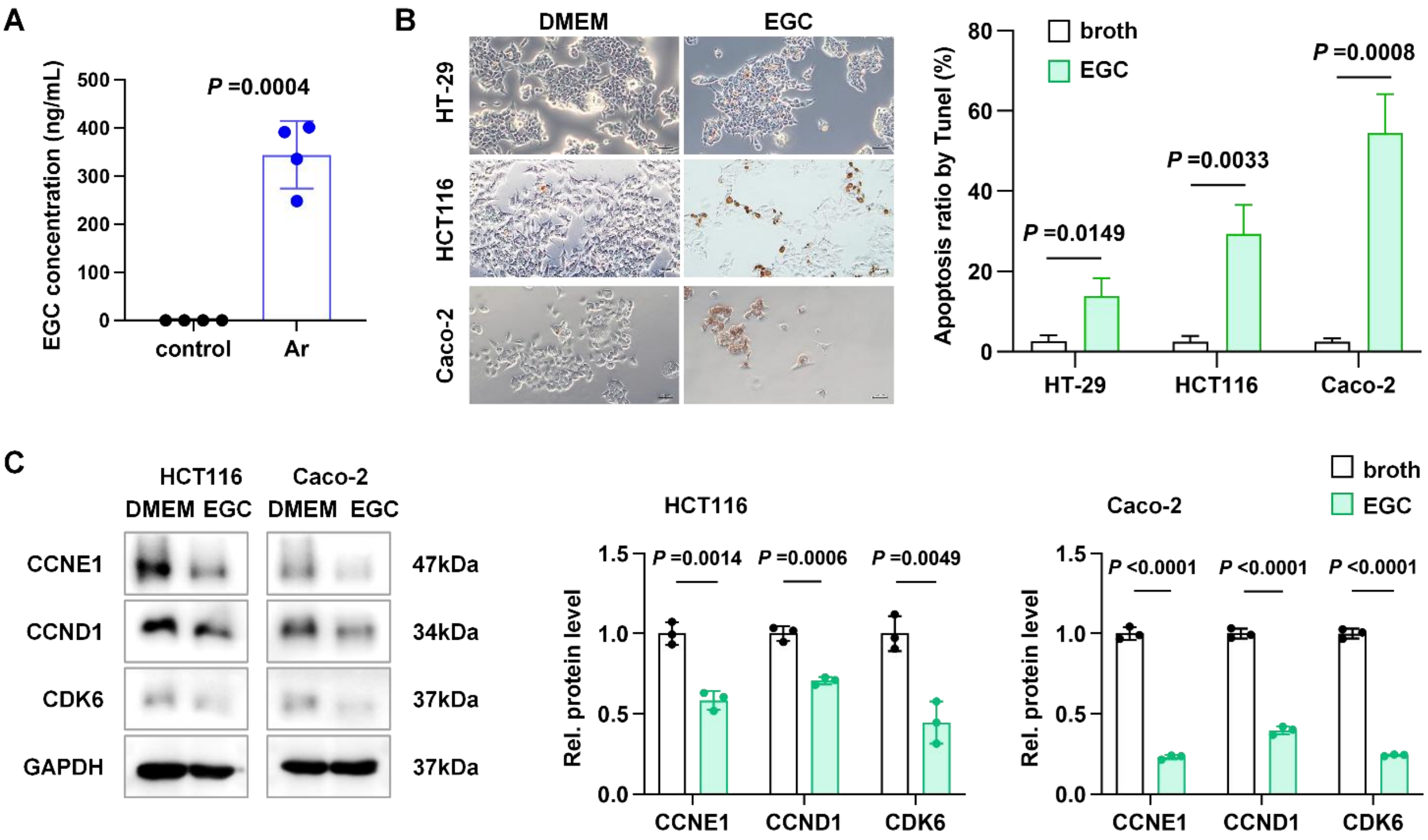

Supplementary Figure 4.

A. EGC levels in control medium and A. rectalis culture supernatants were quantified by targeted LC–MS/MS. Each dot represents an individual biological replicate.

B. CRC cells were treated with DMEM or EGC, followed by TUNEL staining. Representative images and quantification of TUNEL-positive cells are shown.

C. CRC cells were treated with DMEM or EGC, and the indicated proteins were detected by Western blot. GAPDH was used as the loading control. Quantification is shown on the right.

Figure S5

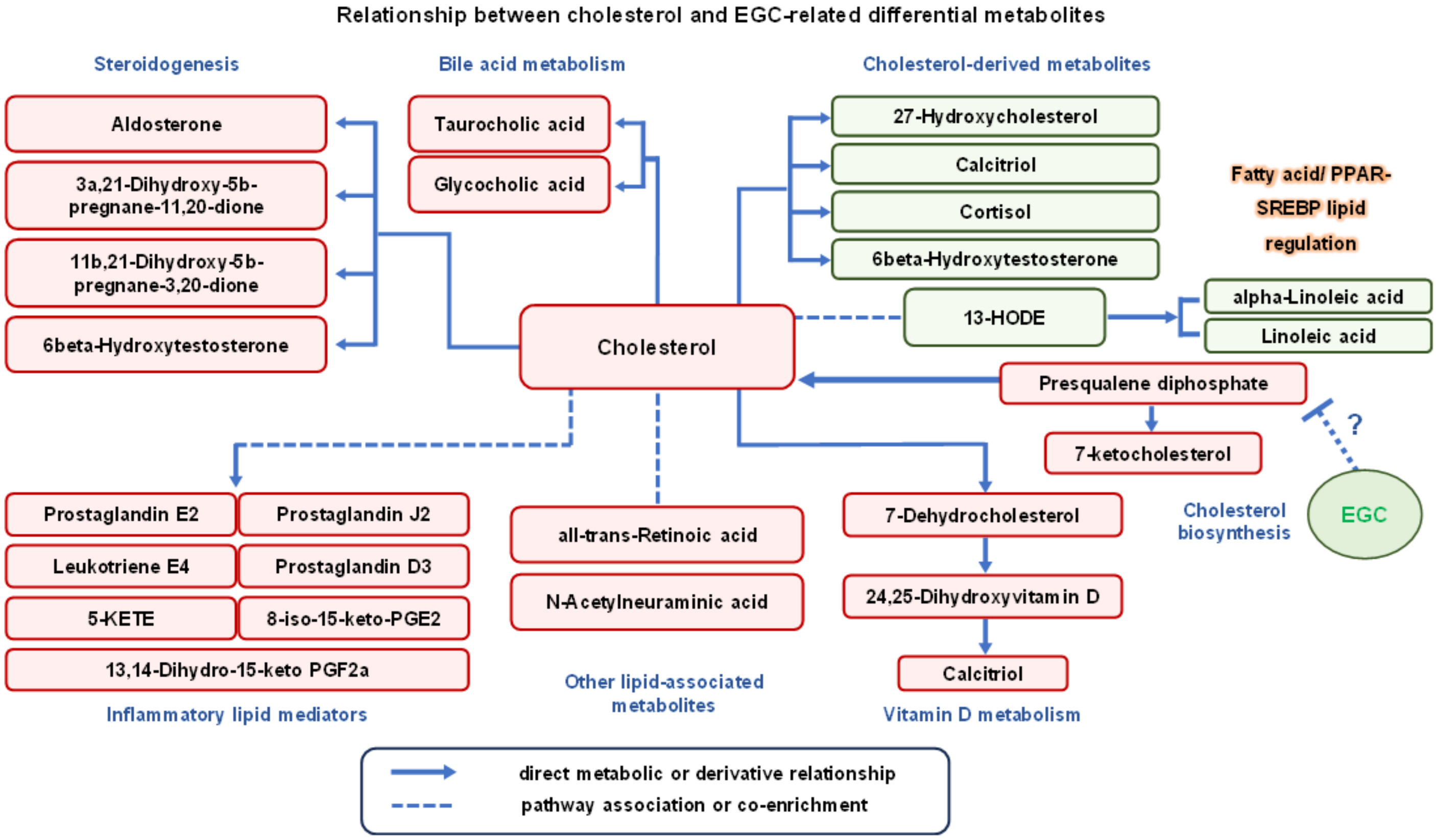

Supplementary Figure 5.

EGC-associated differential metabolites were mapped to cholesterol-derived and lipid-related pathways. Green boxes indicate highlighted metabolites in the EGC-treated group; solid arrows show direct metabolic relationships, and dashed lines indicate pathway association or co-enrichment.

Figure S6

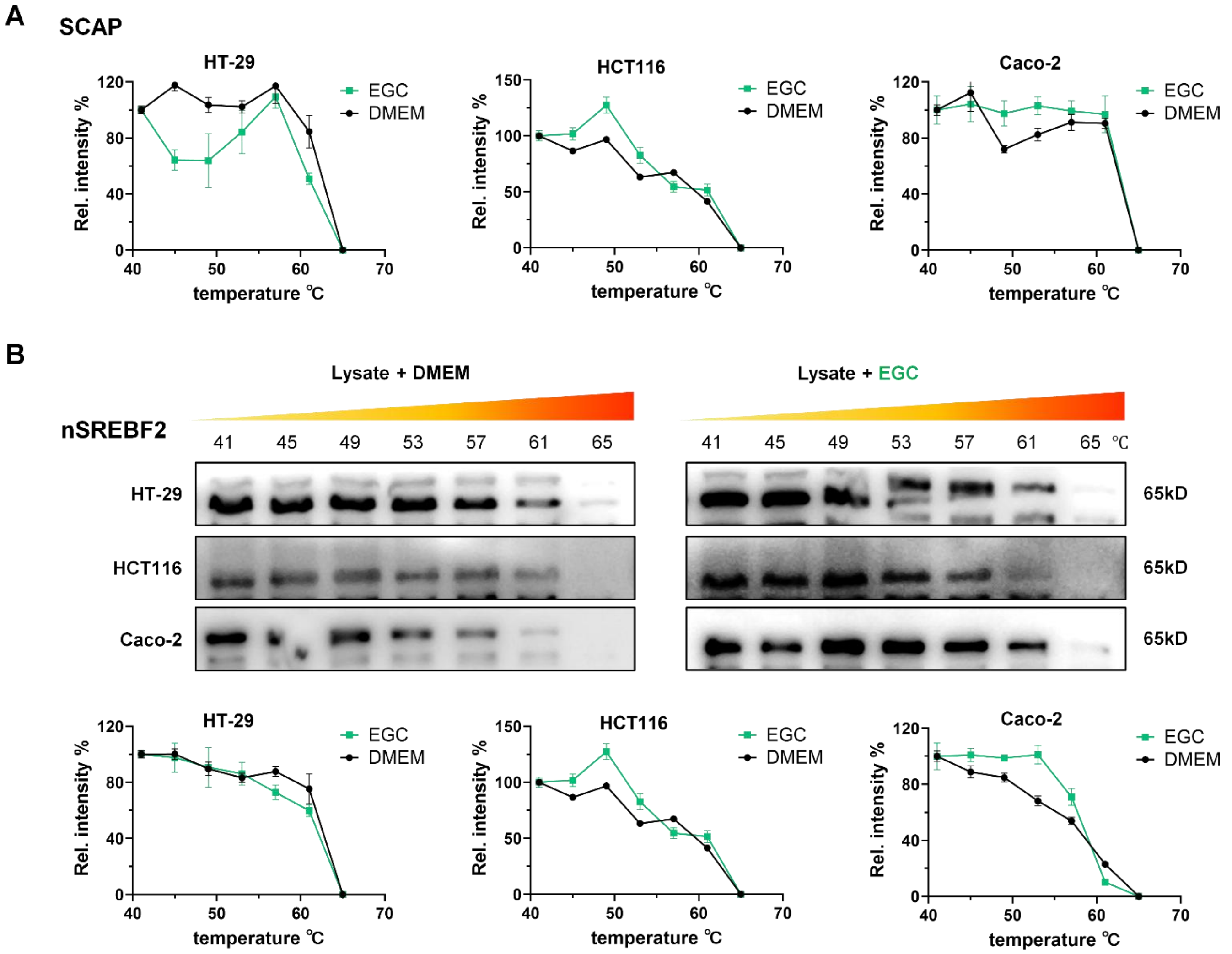

Supplementary Figure 6.

A. Cellular thermal shift assays indicated that EGC does not interact with SCAP.

B. Cellular thermal shift assays indicated that EGC does not interact with nuclear SREBF2 (nSREBF2).

Figure S7

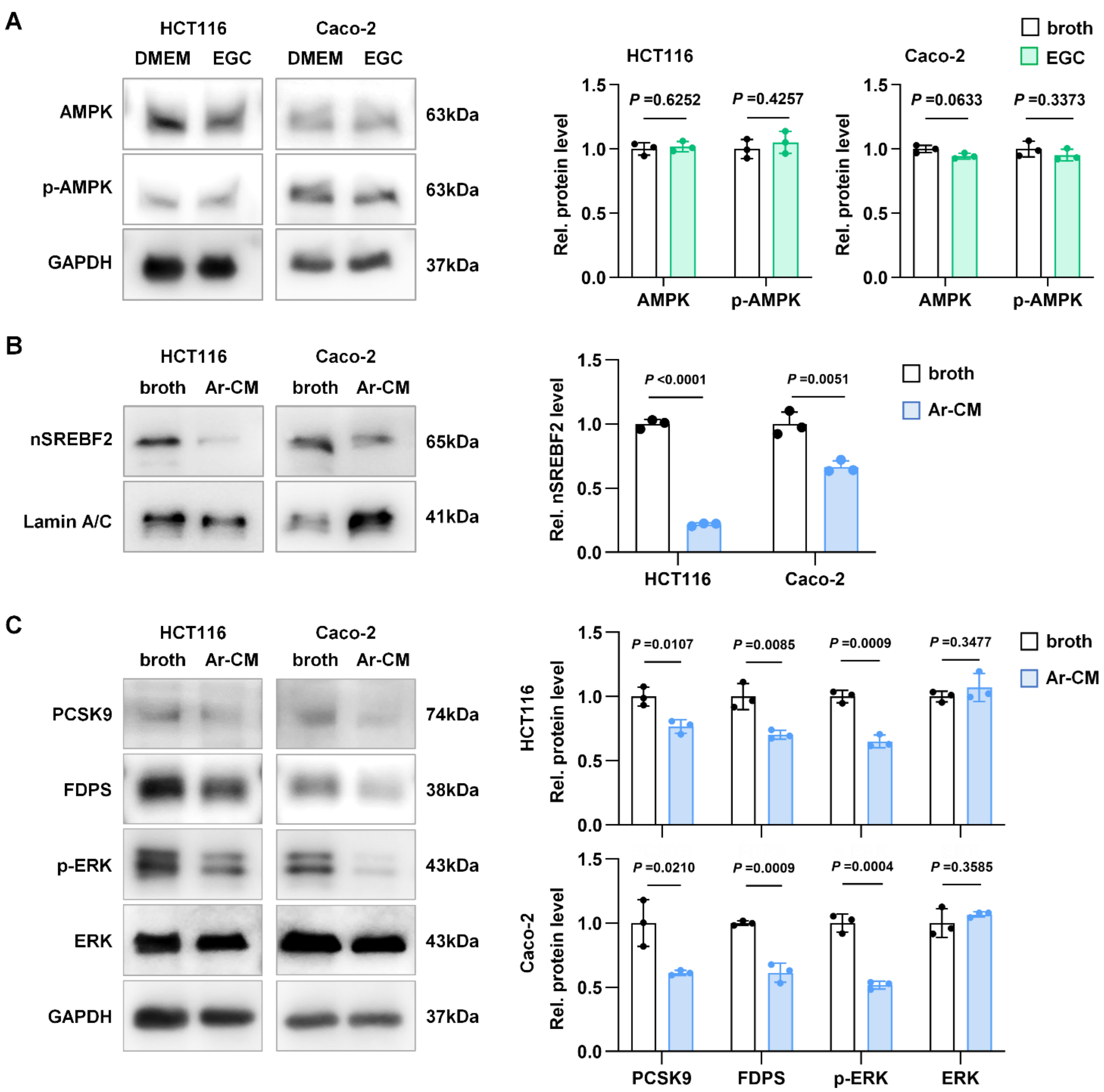

Supplementary Figure 7.

A. CRC cells were treated with DMEM or EGC, and the AMPK/ p-AMPK were detected by Western blot. GAPDH was used as the loading control. Quantification is shown on the right.

B. Western blot analysis showed that Ar-CM reduced nuclear SREBF2 levels, Lamin A/C was used as loading controls.

C. Western blot analysis showed that Ar-CM decreased PCSK9, FDPS, and p-ERK expression in HCT116 and Caco-2 cells, while total ERK remained unchanged. GAPDH were used as loading controls.

Figure S8

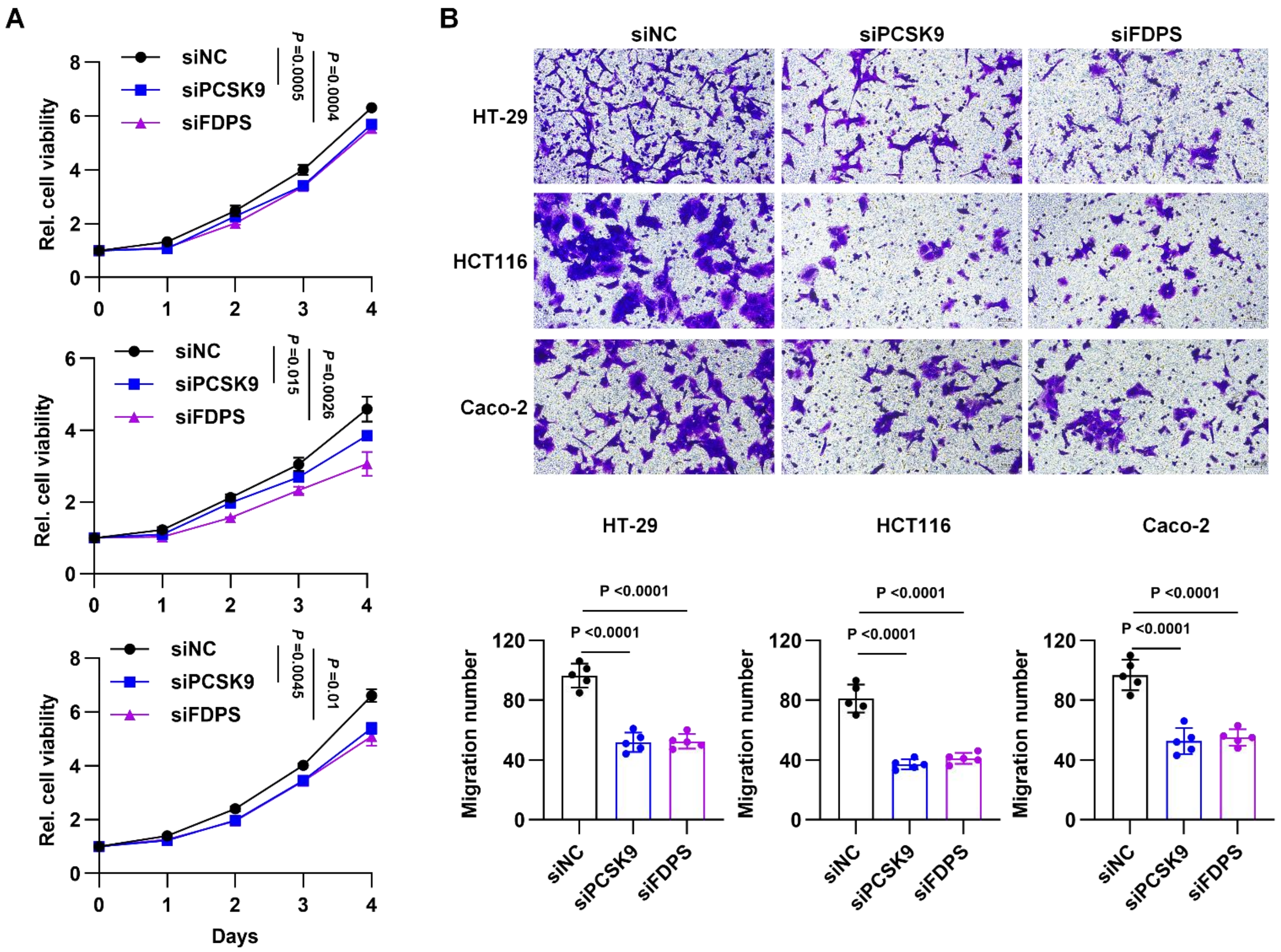

Supplementary Figure 8.

A. Cell viability of HT-29, HCT116, and Caco-2 cells after transfection with si-NC, si-PCSK9, or si-FDPS.

B. Representative transwell migration images and quantification of migrated cells in CRC cells after siRNA-mediated knockdown of PCSK9 or FDPS. Both si-PCSK9 and si-FDPS significantly decreased CRC cell migration.

Figure S9

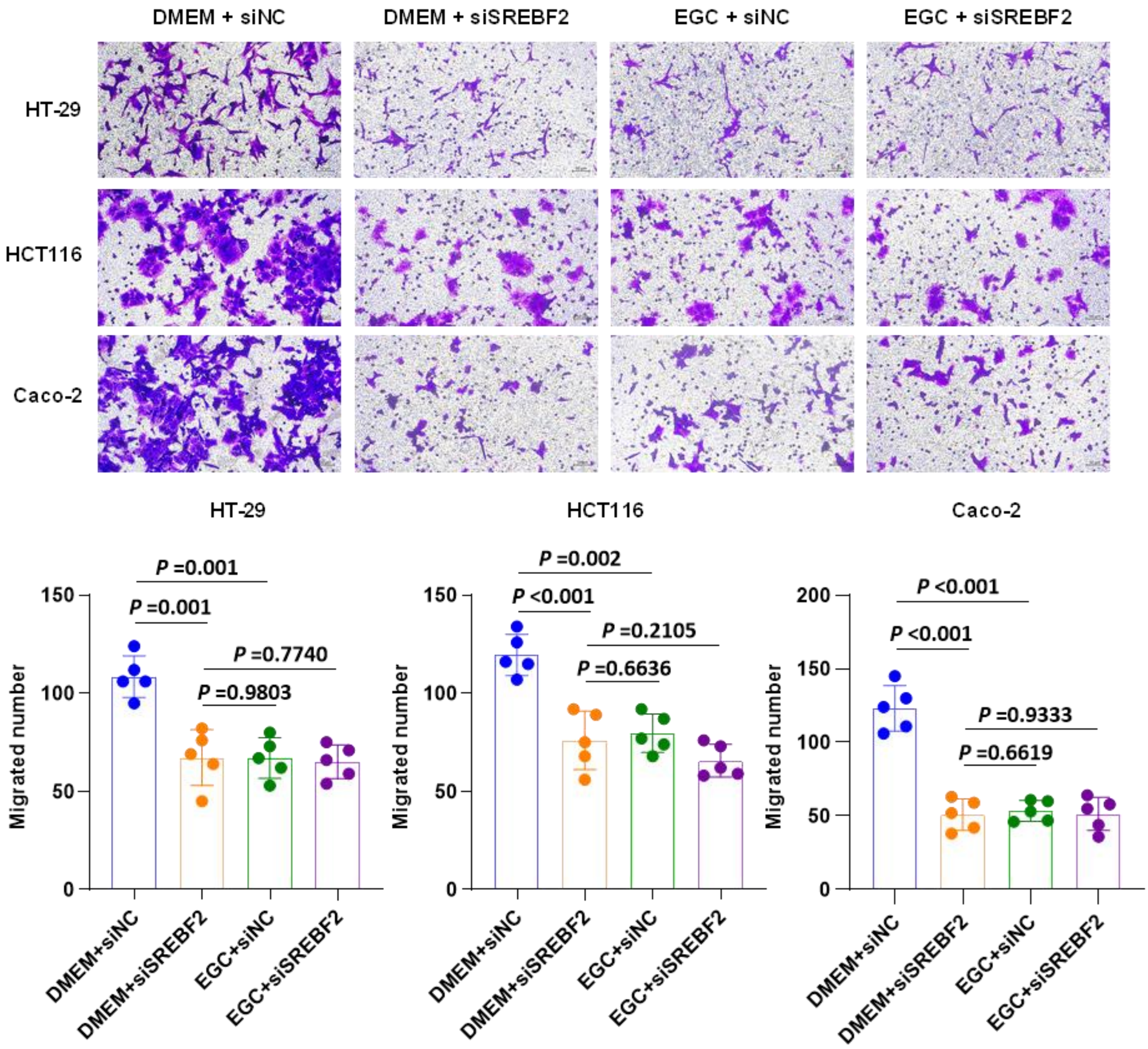

Supplementary Figure 9.

SREBF2 knockdown suppressed CRC cell migration and prevented further EGC-mediated inhibition.
