# Supplementary material for: Agathobacter rectalis suppresses colorectal tumorigenesis via an epigallocatechin–SREBF2 axis controlling cholesterol metabolism
Source: Gut Microbes. 2026 Aug 28;18(1):2724227. doi: 10.1080/19490976.2026.2724227 (PMC13531980; doi:10.1080/19490976.2026.2724227)
Supplement: Supplementary file 2 [file KGMI_A_2724227_SM1543.pdf]

Agathobacter rectalis suppresses colorectal tumorigenesis via an epigallocatechin–SREBF2 axis controlling cholesterol metabolism

Running title: *A. rectalis* inhibits CRC via EGC–SREBF2 axis

Jiawei Lu[1*], Yuting Sun[1*], Yao Zeng[1], Effie Yin Tung Lau[1], Zhien Lan[2], Silin Ye[1], Rui Zhang[1], Ruoyu Hu[1], Chunhui Cui[2], Jessie Qiaoyi Liang[1#]

[1]Department of Medicine and Therapeutics, Li Ka Shing Institute of Health Sciences, CUHK Shenzhen Research Institute, The Chinese University of Hong Kong, Hong Kong, China

[2]Department of General Surgery, Zhujiang Hospital, Southern Medical University, Guangzhou, Guangdong 510280, China

* These authors contributed equally to this work

[#]Correspondence

Dr. Jessie Qiaoyi Liang, Department of Medicine and Therapeutics, The Chinese University of Hong Kong, Shatin, NT, Hong Kong. Email: JessieQY@cuhk.edu.hk; Tel: (852) 3763 6124.

Conflicts of Interest: The authors declare no potential conflicts of interest.

SUPPLEMENTARY METHODS

Sample Processing and Metagenome data analysis

Genomic DNA was extracted using the DNeasy PowerSoil Pro Kit (Qiagen, Hilden, Germany) according to the manufacturer's instructions. The quality and quantity of the extracted DNA were assessed using a NanoDrop 2000 spectrophotometer (Thermo Fisher Scientific, Wilmington, DE) and a Qubit™ Flex Fluorometer with the Qubit™ dsDNA BR Assay Kit (Invitrogen). Sequencing libraries were constructed using the Illumina® DNA Prep kit (Illumina, San Diego, CA) with a standardized DNA input of 300 ng per sample. The resulting libraries were pooled and quantified using the Qubit™ dsDNA HS Assay Kit (Invitrogen), and their integrity was further verified using an Agilent 2100 Bioanalyzer with the High Sensitivity DNA Assay. Finally, libraries were diluted to a starting concentration of 2.5 nM. Metagenomic sequencing was performed on the Illumina NovaSeq 6000 platform for human fecal samples and the NovaSeq X Plus platform for mouse fecal samples.

Stool DNA extraction and species-specific qPCR

Fecal samples were collected from *Apc*$^{Min/+}$ mice at the indicated time points and stored at −80°C until DNA extraction. Microbial genomic DNA was extracted using the BayBiopure Magnetic Bead-Based Fecal Nucleic Acid Extraction Kit (STNM, Baybio, Guangzhou, China) according to the manufacturer's instructions. The abundance of Ar was quantified by species-specific quantitative PCR using primers and a probe targeting Ar. Total bacterial abundance was quantified using universal 16S rRNA gene

primers/probe and used for normalization. qPCR was performed using Premix Ex Taq™ (RR390, Takara, Osaka, Japan) on on the QuantStudio 7 Flex System (Applied Biosystems, Carlsbad, CA, USA). Each reaction was performed in technical triplicate. Statistical analysis was performed using an unpaired two-tailed Student's t-test for two-group comparisons and one-way ANOVA followed by multiple-comparison correction for comparisons among multiple time points. A value of $P < 0.05$ was considered statistically significant. Primers and probes used in this study are as follows: Ar-F: GGTTTTCGTCCTCCGGAACT; Ar-R: CAGGCAACGTTAAGTGAACATATCA; Ar-probe (5'-TAMRA and 3'-FAM): CCGATAGAAGTCTCATCCAACTCGCCTGAC.

Metagenomic profiling and microbial diversity analysis

Species-level microbial abundance profiles were generated from metagenomic sequencing data using MetaPhlAn. For alpha-diversity and beta-diversity analysis, species-level relative abundance data were extracted from the merged abundance table, and taxa with zero abundance across all selected samples were removed. Stools from F. nucleatum-colonized $Apc^{Min/+}$ mice treated with PBS and Ar for 12 weeks as indicated in Figure 1B were included for comparison. Alpha-diversity indices, including the Shannon index, Simpson index, inverse Simpson index, and observed species richness, were calculated based on the normalized species-level microbial abundance matrix. Differences between the control and Ar groups were assessed using the Wilcoxon rank-sum test. Alpha-diversity plots were generated using R and GraphPad Prism. Bray–Curtis dissimilarity was calculated based on the normalized

species-level relative abundance matrix using the vegan package in R. Principal coordinate analysis (PCoA) was performed to visualize differences in microbial community structure between groups. Statistical significance of group separation was evaluated by permutational multivariate analysis of variance (PERMANOVA) using the adonis2 function with 999 permutations. PCoA plots were generated using the ggplot2 package in R.

Annexin V/PI apoptosis assay

CRC cells were seeded into 6-well plates and treated with control broth or Ar-conditioned medium for 48 hours. After treatment, both floating and adherent cells were collected, washed twice with cold PBS, and resuspended in binding buffer. Cells were then stained with Annexin V and propidium iodide according to the manufacturer's instructions. Apoptotic cells were detected by flow cytometry. Annexin V-positive/PI-negative cells were defined as early apoptotic cells, whereas Annexin V-positive/PI-positive cells were defined as late apoptotic cells. Total apoptosis was calculated as the sum of early and late apoptotic populations.

TUNEL staining assay

CRC cells were seeded onto 24-well plates and treated with DMEM or EGC for 48 hours. Cells were fixed with 4% paraformaldehyde, permeabilized, and subjected to TUNEL staining according to the manufacturer's protocol. TUNEL-positive cells were visualized under a light microscope. Images were captured from randomly selected

fields, and the percentage of apoptotic cells was calculated as the number of TUNEL-positive cells divided by the total number of cells. Quantification was performed from at least three independent fields per group.

Cholesterol Quantification Assay

Cholesterol Quantification Assay was performed using a commercial kit (BC1985, Solarbio, Beijing, China). Thirty milligrams of feces are accurately weighed and combined with 400 mL of extraction solution. The mixture is homogenized in an ice bath and then sonicated in an ice bath. Subsequently, the mixture is centrifuged at 10,000 g and 4°C for 10 minutes, and the supernatant is kept on ice for measurement. A 50 μmol/mL cholesterol stock solution is prepared by dissolving 100 mg of cholesterol standard in 517 μL of extraction solution, and the stock solution is diluted with extraction solution to 10, 5, 2.5, 1.25, 0.625, and 0.3175 μmol/mL to generate standard solutions. The working solution is prepared according to the kit instructions and incubated at 37°C for 30 minutes. The working solution is then mixed with the standard solutions, the sample solution, and the control solution. Absorbance is measured at 500 nm using a microplate reader, and the cholesterol content of the samples is calculated from the standard curve.

Cell culture conditions

Colon cancer cell lines HT-29 (RRID: CVCL_0320), HCT116 (RRID: CVCL_0291), and Caco-2 (RRID: CVCL_0025) were obtained from ATCC (Apr 2021). All cells were confirmed to be contamination free and have not been reported as misidentified or contaminated according to the International Cell Line Authentication Committee (ICLAC) database. The STR profile of our cells showed a 100% match with the ATCC

reference database. Cells were maintained in Dulbecco's Modified Eagle Medium (DMEM; Gibco, Waltham, MA) supplemented with 10% fetal bovine serum (FBS; Gibco) and 1% penicillin–streptomycin (Gibco), at 37°C in a humidified 5% CO2 incubator. Cells were pretreated with one of the following conditions prior to subsequent experiments: 5% culture supernatant from Ar or E. coli, 5% YCFA, 80 μM EGC, or DMEM.

Untargeted liquid chromatography–tanden mass spectrometry (LC–MS/MS)-based metabolomic analysis

Culture supernatants (100 μL) were combined with 300 μL of extraction solvent (methanol containing an isotopically labeled internal standard mix), vortexed for 30 s, sonicated in an ice–water bath for 10 min, and then incubated at −40°C for 1 h to precipitate proteins. Samples were centrifuged at 12,000 rpm for 15 min at 4°C, and the supernatants were transferred to clean glass vials for analysis.

LC–MS/MS was performed on a UHPLC system (Vanquish, Thermo Fisher Scientific, Waltham, MA) equipped with a UPLC HSS T3 column (2.1 × 100 mm, 1.8 μm) and coupled to an Orbitrap Exploris 120 mass spectrometer (Thermo Fisher Scientific). Mobile phase A was water with 5 mM ammonium acetate and 5 mM acetic acid; mobile phase B was acetonitrile. The autosampler was held at 4°C, and 2 μL was injected per run. The Orbitrap Exploris 120 acquired MS/MS spectra in information-dependent acquisition mode using Xcalibur (Thermo Fisher Scientific). ESI settings were: sheath gas 50 Arb, auxiliary gas 15 Arb, capillary temperature 320°C, full MS resolution 60,000, MS/MS resolution 15,000, normalized collision energies 10/30/60, and spray voltage 3.8 kV (positive mode) or −3.4 kV (negative mode).

Raw data were converted to mzXML with ProteoWizard and processed in R using an XCMS-based workflow (Biotree, Shanghai, China) for peak detection, extraction, alignment, and integration. Metabolite annotations were assigned using MS2 spectral matches against BiotreeDB (Biotree), the Human Metabolome Database (HMDB; www.hmdb.ca), and the METLIN metabolite database (metlin.scripps.edu).

Metabolic pathway analysis

Metabolic pathway analysis was performed using MetaboAnalyst 6.0 (https://www.metaboanalyst.ca/) based on the differentially abundant metabolites identified in the metabolomic profiling. Prior to pathway analysis, metabolite names were manually checked and standardized to improve database matching. Differential metabolites were uploaded to MetaboAnalyst for metabolite set enrichment analysis and pathway enrichment analysis using the pathway libraries implemented in the platform. Pathway enrichment significance was evaluated using enrichment analysis, and multiple testing correction was performed using the Benjamini–Hochberg false discovery rate (FDR) method. Enriched pathways were ranked according to enrichment score, FDR value, and the number of matched metabolites. Pathways related to cholesterol and lipid metabolism were further examined in detail. The involved differential metabolites were then manually mapped to their corresponding metabolic branches according to the enriched pathway annotations. Integrated pathway schematics were generated to visualize direct metabolic or derivative relationships and pathway association/co-enrichment relationships among the altered metabolites.

Growth curve assay

Cell viability assay was measured using the Enhanced Cell Counting Kit-8 (CCK-8; Beyotime, Nantong, China) for three or four consecutive days. The absorbance was measured at OD450 using a CLARIOstarplus microplate reader (BMG LABTECH, Ortenberg, Germany).

Colony formation assay

Cells were plated in 6-well dishes at 1,000 cells per well and allowed to attach for 24 h before initiating treatments. Media were replaced every three days. After three weeks of treatment, cells were fixed with 4% paraformaldehyde and stained with 0.1% crystal violet. Colonies containing more than 50 cells were counted.

Transwell assay

Cells ($5*10^4$) suspended in serum-free DMEM were seeded in the upper chamber of the Matrigel Invasion chambers (BD Biosciences, San Jose, CA). DMEM with 20% FBS was added to the wells below. After 72 h culture, cells were fixed with 4% paraformaldehyde and stained with 0.1% crystal violet.

Flow cytometry

For cell cycle analysis, cells were seeded into 6-well plates and allowed to attach for 24 h. They were then switched to serum-free DMEM and incubated for another 24 h, followed by a 24 h treatment with either Ar supernatant or broth. Cells were collected, fixed in 75% ice-cold ethanol at 4°C overnight, and stained with PI–RNase solution (BD Biosciences) for 30 min at room temperature in the dark. Fluorescence was

acquired on a CytoFlex LX flow cytometer (Beckman Coulter, Miami, FL), and cell cycle phases were modeled using ModFit v3.0 (Verity Software House, Topsham, ME).

The half maximal inhibitory concentration (IC_{50}) assay

Cells in the logarithmic growth phase were seeded into 96-well plates, then incubated at 37°C with 5% CO2 for 24 h to allow attachment. An EGC concentration gradient was prepared by serial 1:10 dilutions. The medium was replaced with complete medium containing the indicated EGC concentrations and cells were treated for 48 h. Cell viability was assessed using the CCK-8 assay by adding 10% of the well volume of CCK-8 reagent and incubating at 37°C for 1 h, followed by absorbance reading at 450 nm.

RNA extraction and sequencing

Total RNA was extracted from cell lines using TRIzol reagent (Invitrogen, Carlsbad, CA). Then RNA quality was determined by 5300 Bioanalyser (Agilent) and quantified using the ND-2000 (NanoDrop Technologies). RNA purification, reverse transcription, library construction and sequencing were performed at Shanghai Majorbio Bio-pharm Biotechnology Co., Ltd. (Shanghai, China) according to the manufacturer's instructions. The RNA-seq transcriptome library was prepared following Illumina Stranded mRNA Prep, Ligation (San Diego,CA) using 1μg of total RNA. After quantified by Qubit 4.0, the sequencing library was performed on DNBSEQ-T7 platform (PE150) using DNBSEQ-T7RS Reagent Kit (FCL PE150) version 3.0. To identify DEGs (differential expression genes) between two different samples, the expression level of each transcript was calculated according to the transcripts per million reads (TPM) method. Differential expression analysis was conducted using

DESeq in the Majorbio Cloud (www.majorbio.com).

Pathway analysis

Differentially expressed genes were identified from normalized expression data using, applying an adjusted P value (Benjamini–Hochberg FDR) <0.2 and an absolute log2 fold change ≥ 0.15 as significance thresholds. Functional enrichment was performed for Wikipathways using over‐representation analysis (Fisher's exact test) implemented in WEB-based GEne SeT AnaLysis Toolkit (https://www.webgestalt.org/). Pathway networks analysis was performed in R (version 4.5.2) via R packages (dplyr, stringr, igraph, tidygraph, ggraph, ggplot2, cowplot, grid, openxlsx). Pathway similarity network was constructed by computing Jaccard similarity indices between each pair of pathway gene sets. code and parameters are provided in Github (https://github.com/JarethLu/EGCpathway) to ensure reproducibility.

Targeted metabolomics analysis

EGC was extracted from samples using a protein precipitation method. Briefly, 200 μL of each sample was accurately transferred into a centrifuge tube, followed by the addition of two volumes of ethanol containing 0.1% formic acid. The mixture was vortexed thoroughly and incubated at −20°C for 1 h to precipitate proteins. After centrifugation at 12,000 rpm for 10 min, the supernatant was collected for LC–MS/MS analysis. The extracts were analyzed using a Triple Quad 4500 LC–MS/MS System (AB Sciex, Beijing, China) equipped with a Shim-pack GIST-HP HSS column (2.1 × 150 mm). The flow rate was set at 0.45 mL/min. The mobile phase consisted of 0.1% formic acid in water as solvent A and acetonitrile containing 0.1% formic acid as solvent B. Standard curves for accurate concentration determination were established

using serial dilutions of EGC standard samples prepared in methanol. Quality control samples were included in each analytical batch to ensure the reliability and reproducibility of the measurements.

Histochemistry and Immunohistochemistry

Primary antibodies used in this study include: anti-Ki67 (9129, CST, Beverly, MA), anti-ERK (11257-1-AP, Proteintech, Rosemont, IL), anti- phospho-ERK (28733-1-AP, Proteintech), anti-FDPS (ab153805, Abcam, Cambridge, UK), anti-PCSK9 (27882-1-AP, Proteintech). Secondary antibodies used in this study include: Goat Anti-Rabbit IgG H&L (HRP) (ab6721, Abcam), Goat Anti-Mouse IgG (H+L) (HRP) (ABclonal, Wuhan, China). Primary and secondary antibodies were used with 1:400 and 1:1000 dilutions, respectively.

SREBF2 transcriptional activity assay

Complete Transcription Factor Binding (CTFB) Assay Buffer was prepared in accordance with the manufacturer's Pre‑Assay Preparation guidelines and dispensed into designated blank (Blk) and nonspecific binding (NSB) wells. Where applicable, competitor double‑stranded DNA (dsDNA) and the supplied positive control were added to the appropriate wells, followed by addition of samples containing SREBP‑2. Plates were incubated overnight at 4°C without agitation to permit binding. Subsequently, wells were washed five times with 1× Wash Buffer. A diluted SREBP‑2 primary antibody was applied to all wells except Blk wells and incubated at ambient temperature without agitation, after which plates were washed an additional five times with 1× Wash Buffer. A diluted goat anti‑rabbit horseradish peroxidase (HRP)–

conjugated secondary antibody was then added to all wells except Blk wells and incubated at ambient temperature without agitation, followed by five further washes with 1× Wash Buffer. Developing Solution was added and incubated for 15–45 minutes with gentle agitation, and the reaction was quenched with Stop Solution. Absorbance was measured at 450 nm.

Patient-derived organoid culture and treatment

Fresh colorectal tumor tissue was obtained from one patient undergoing surgical resection after written informed consent and approval by the institutional ethics committee. The patient was a 67-year-old female diagnosed with colorectal adenocarcinoma located in the rectum, with pathological stage T4N1bMx. Tumor involvement of the anal epithelium was observed. Lymphovascular invasion was present, while no definite perineural invasion was identified. The tumor tissue was processed immediately after surgical resection for organoid establishment. Briefly, the tumor tissue was washed with cold PBS, mechanically minced into small fragments, and enzymatically digested to isolate tumor epithelial clusters. The isolated cell clusters were embedded in Matrigel and cultured in colorectal cancer organoid medium under standard conditions. After successful establishment and expansion, organoids were used for treatment experiments. For each independent experiment, 3 technical replicate wells were included for each treatment condition. Image quantification was performed by an investigator blinded to the treatment groups.

Cellular thermal shift assay (CETSA)

Cells were grown to the desired density, harvested, and washed with ice-cold PBS. Cells were resuspended in PBS supplemented with a complete protease inhibitor cocktail. Cell suspensions were lysed by three liquid nitrogen freeze–thaw cycles and clarified by centrifugation at 20,000 × g for 20 minutes at 4°C to obtain the soluble fraction. Lysates were diluted as required and divided into two portions: one was treated with EGC and the other with DMEM (vehicle control), followed by incubation at room temperature for 30 minutes. Each condition was then aliquoted into 50 μL portions and subjected to a temperature gradient predefined in preliminary CETSA experiments, with heating for 3 minutes and subsequent cooling for 3 minutes at room temperature. Samples were centrifuged again at 20,000 × g for 20 minutes at 4°C to separate heat-precipitated proteins from the soluble fractions. The supernatants were transferred to fresh tubes and analyzed by SDS-PAGE followed by Western blotting to quantify the soluble target protein across temperatures.

Protein extraction and western blotting

Primary antibodies used in this study include: anti-SREBF2 (28212-1-AP, Proteintech), anti-ERK (11257-1-AP, Proteintech, Rosemont, IL), anti- phospho-ERK (28733-1-AP, Proteintech), anti-FDPS (ab153805, Abcam, Cambridge, UK), anti-PCSK9 (27882-1-AP, Proteintech), anti- Lamin A+ Lamin C (ab108595, Abcam), SCAP (anti-SCAP, ab125186, Abcam) anti- GAPDH (D110016, Sangon, Shanghai, China), anti-AMPK (2532S; CST), anti-pAMPK (2535S; CST), anti-CCND1 (2922S; CST), anti-CCNE1 (20808S; CST), anti-CDK6 (13331S; CST). Secondary antibodies used in this study include: Goat Anti-Rabbit IgG H&L (HRP) (ab6721, Abcam, Cambridge, UK), Goat Anti-Mouse IgG (H+L) (HRP) (ABclonal, Wuhan, China). Primary and secondary antibodies were used with 1:1000 and 1:10000 dilutions, respectively.

Co-Immunoprecipitation (Co-IP)

Co-IP was performed using the Co‑IP Kit (HY‑K0202K, MedChemExpress) in accordance with the manufacturer's instructions. Cells were washed twice with 1× PBS, detached with a cell scraper, and transferred to 1.5‑mL microtubes. Lysis buffer was added at 25 μL per 1 × 10^5 cells together with PMSF and a protease inhibitor cocktail, mixed thoroughly, and incubated on ice for 10 min. Lysates were clarified by centrifugation (14,000 × g, 10 min, 4°C), and supernatants were kept on ice or stored at −20°C. For bead preparation, 25 μL of magnetic protein A/G beads were fully resuspended, washed three times with 200 μL binding/wash buffer using a magnetic rack, and the supernatants were discarded. For each sample, 5 μg of primary antibody (anti-SCAP, ab125186, Abcam) was added to the cell lysate and the mixture was diluted with binding/wash buffer to a total volume of 500 μL. Samples were incubated on a rotator (room temperature for 1–2 h or 4°C overnight) to form antigen–antibody complexes. The immune complexes were then incubated with the pre-equilibrated magnetic beads for 4 h at 4°C with rotation, followed by magnetic separation to collect the beads; supernatants were retained for optional analyses. Beads were washed three times with 500 μL binding/wash buffer, each time mixing gently and separating magnetically, and supernatants were collected if needed. Bound proteins were eluted by adding 100 μL of 1× SDS sample buffer, heating at 95°C for 5 min, and magnetically separating the beads; eluates were analyzed by SDS–PAGE using a pre-stained protein marker and subsequent immunoblotting.
