# Supplementary material for: Agathobacter rectalis suppresses colorectal tumorigenesis via an epigallocatechin–SREBF2 axis controlling cholesterol metabolism
Source: Gut Microbes. 2026 Aug 28;18(1):2724227. doi: 10.1080/19490976.2026.2724227 (PMC13531980; doi:10.1080/19490976.2026.2724227)
Supplement: Supplementary file 3 [file KGMI_A_2724227_SM5146.pdf]

NOTE: Please save this file locally before filling in the table, DO NOT work on the file within your internet browser as changes will not be saved. Adobe Acrobat Reader (available free here) is recommended for completion.

The ARRIVE guidelines 2.0: author checklist

The ARRIVE Essential 10

These items are the basic minimum to include in a manuscript. Without this information, readers and reviewers cannot assess the reliability of the findings.

Item		Recommendation	Section/line number, or reason for not reporting
**Study design**	1	For each experiment, provide brief details of study design including:<br>a. The groups being compared, including control groups. If no control group has been used, the rationale should be stated.<br>b. The experimental unit (e.g. a single animal, litter, or cage of animals).	In the Apcmin/+ mouse model, mice were gavaged with Ar. E. coli and PBS served as negative controls, to investigate and compare effects on tumor development of the bacteria.<br><br>Mice were housed according to their experimental groups. The housing density did not exceed 5 mice per standard cage, or 10 mice when a larger cage was used.
**Sample size**	2	a. Specify the exact number of experimental units allocated to each group, and the total number in each experiment. Also indicate the total number of animals used.<br>b. Explain how the sample size was decided. Provide details of any *a priori* sample size calculation, if done.	The sample size for each mouse model was as follows:<br>• In the 1st Apcmin/+ mouse model of figure 1: There were 5 mice in E. coli group, 9 mice in PBS group, 10 mice in the Ar1 group and 8 mice in the Ar2 group, totaling 32 mice.<br>• In 2nd Apcmin/+ mouse model of figure 4: There were 9 mice PBS group, and 8 mice in EGC group, totaling 17 mice.<br><br>Sample sizes were chosen based on our lab's established experience and common practice in the field, as preliminary data for a formal power calculation were unavailable. We selected group sizes that balanced statistical needs with ethical (3Rs) and practical considerations. This led to 9-12 mice per group for the Apcmin/+ models.
**Inclusion and exclusion criteria**	3	a. Describe any criteria used for including and excluding animals (or experimental units) during the experiment, and data points during the analysis. Specify if these criteria were established *a priori*. If no criteria were set, state this explicitly.<br>b. For each experimental group, report any animals, experimental units or data points not included in the analysis and explain why. If there were no exclusions, state so.<br>c. For each analysis, report the exact value of *n* in each experimental group.	No formal, a priori inclusion or exclusion criteria were pre-defined for this study. However, animals were monitored daily, and any that met humane endpoints (e.g., severe lethargy, > 20% body weight loss, or signs of irreversible distress) were euthanized and excluded from the final analysis. All data points were included in the analysis without exclusion.<br><br>Mice were monitored daily and all data points were included in the analysis without exclusion.<br><br>• Apcmin/+ model 1: n = 10 for the Ar1, n=8 for the Ar2; n = 5 for E. coli, and n=9 for PBS groups.<br>• Apcmin/+ model 2: n = 8 for the EGC, n = 9 for the PBS group.
**Randomisation**	4	a. State whether randomisation was used to allocate experimental units to control and treatment groups. If done, provide the method used to generate the randomisation sequence.<br>b. Describe the strategy used to minimise potential confounders such as the order of treatments and measurements, or animal/cage location. If confounders were not controlled, state this explicitly.	Mice were randomly assigned to groups by drawing lots with no significant difference in initial body weight.<br><br>Animals were randomly allocated to all experimental groups to distribute potential unknown confounding variables evenly across groups.
**Blinding**	5	Describe who was aware of the group allocation at the different stages of the experiment (during the allocation, the conduct of the experiment, the outcome assessment, and the data analysis).	Due to the nature of the treatments, personnel responsible for animal care and treatment administration (e.g., oral gavage) were unavoidably aware of the group allocations. However, to minimize bias, all outcome assessments were performed in a blinded manner. This included tumor measurement (e.g., with calipers), histological analysis, and marker quantification. The blinding was achieved by using ear tags that concealed the treatment group identity.
**Outcome measures**	6	a. Clearly define all outcome measures assessed (e.g. cell death, molecular markers, or behavioural changes).<br>b. For hypothesis-testing studies, specify the primary outcome measure, i.e. the outcome measure that was used to determine the sample size.	Primary Outcome Measures:<br>• Tumor load: The total number and volume of tumors per mouse (assessed in the Apcmin/+ models)..<br>Secondary Outcome Measures:<br>• Molecular Marker Levels: Quantification of specific markers (e.g., the proliferation marker Ki-67) in tumor tissue via immunohistochemistry.<br>• Pathological Assessment: Evaluation of tissue sections for incidence of dysplasia.<br>• Colon length: Evaluate the colon length from anus to cecum by calipers<br><br>The primary outcome measure for hypothesis testing was tumor load. This was quantified as either tumor size or tumor number per mouse for the purpose of sample size determination.
**Statistical methods**	7	a. Provide details of the statistical methods used for each analysis, including software used.<br>b. Describe any methods used to assess whether the data met the assumptions of the statistical approach, and what was done if the assumptions were not met.	All statistical tests were performed using Graphpad Prism 9.4.1 (Graphpad Software Inc., San Diego, CA) or MedCalc Statistical Software version 22.021 (MedCalc Software Ltd, Ostend, Belgium). Statistical significance was defined as a two-sided P-value of < 0.05.<br><br>The choice of statistical test was guided by diagnostic checks of the data. For multiple group comparisons, we used ordinary one-way ANOVA with Tukey's test if parametric assumptions were satisfied. All
**Experimental animals**	8	a. Provide species-appropriate details of the animals used, including species, strain and substrain, sex, age or developmental stage, and, if relevant, weight.<br>b. Provide further relevant information on the provenance of animals, health/immune status, genetic modification status, genotype, and any previous procedures.	In Apcmin/+ mouse model, six-week-old Apcmin/+ C57BL/6J male mice were randomized into two groups.<br><br>The Apcmin/+ mice were on a C57BL/6J background and were genotyped before the experiment to confirm the genotype. All animals were specific pathogen-free (SPF) and housed under standard conditions.
**Experimental procedures**	9	For each experimental group, including controls, describe the procedures in enough detail to allow others to replicate them, including:<br>a. What was done, how it was done and what was used.<br>b. When and how often.<br>c. Where (including detail of any acclimatisation periods).<br>d. Why (provide rationale for procedures).	• Apcmin/+ mouse Model:<br>• What & How: Mice were orally gavaged with Ar,, E. coli (containing approximately 108 bacterial cells), 100 uM EGC (200 μL), or PBS (200 μL).<br>• What was used: A daily prepared suspension of either Ar or E. coli MG1655 at a dose of 1×10^9 CFU in 200 μL PBS, with PBS alone as the vehicle control.<br><br>• Apcmin/+ Model 1:<br>o Treatment: Daily gavage for 12 weeks.<br>o Stool Collection: Weekly, 24 hours after the previous gavage.<br><br>• Location: All procedures (gavage, tumor measurements) were performed in a dedicated procedure room within the animal facility<br><br>• Apcmin/+ Model: To chronically assess the oncogenic effects of sustained daily bacterial colonization on gut tumorigenesis.
**Results**	10	For each experiment conducted, including independent replications, report:<br>a. Summary/descriptive statistics for each experimental group, with a measure of variability where applicable (e.g. mean and SD, or median and range).<br>b. If applicable, the effect size with a confidence interval.	

The Recommended Set

These items complement the Essential 10 and add important context to the study. Reporting the items in both sets represents best practice.

Item		Recommendation	Section/line number, or reason for not reporting
**Abstract**	11	Provide an accurate summary of the research objectives, animal species, strain and sex, key methods, principal findings, and study conclusions.	This study aims to investigate the function of Ar in CRC and the underlying mechanisms involved. In Apcmin/+ models, oral gavage of Ar significantly decreases colon tumor incidence, siz
**Background**	12	a. Include sufficient scientific background to understand the rationale and context for the study, and explain the experimental approach.	To comprehensively investigate this, we employed Apcmin/+ model to test the preventive effect of Ar on genetically-driven spontaneous tumorigenesis.
		b. Explain how the animal species and model used address the scientific objectives and, where appropriate, the relevance to human biology.	Apcmin/+ Model directly addresses genetic predisposition, as the Apc gene mutation is a key initiating event in human CRC. It is ideal and the most common use for studying intestinal tumor development.
**Objectives**	13	Clearly describe the research question, research objectives and, where appropriate, specific hypotheses being tested.	This study aims to investigate the function of Ar in CRC and the underlying mechanisms involved.
**Ethical statement**	14	Provide the name of the ethical review committee or equivalent that has approved the use of animals in this study, and any relevant licence or protocol numbers (if applicable). If ethical approval was not sought or granted, provide a justification.	Animal experiments were conducted following the guidelines approved by the Animal Ethics Committee of The Chinese University of Hong Kong (AEEC ref# 22-126-GRF).
**Housing and husbandry**	15	Provide details of housing and husbandry conditions, including any environmental enrichment.	Mice were group-housed under specific pathogen-free (SPF) conditions in individually ventilated cages (IVCs) maintained at a 12-hour light/dark cycle, with ad libitum access to standard ch
**Animal care and monitoring**	16	a. Describe any interventions or steps taken in the experimental protocols to reduce pain, suffering and distress.	All interventions strictly adhered to animal ethics guidelines, incorporating daily health monitoring and predefined humane endpoints (e.g., >20% weight loss or signs of severe distress). All procedures were performed by experienced staff to minimize animal discomfort.
		b. Report any expected or unexpected adverse events.	No obviously adverse events were observed or reported in this study.
		c. Describe the humane endpoints established for the study, the signs that were monitored and the frequency of monitoring. If the study did not have humane endpoints, state this.	Humane endpoints were strictly predefined as severe lethargy, moribund state, or >20% body weight loss from baseline, with all animals monitored daily for these signs and tumor burden throughout the study.
**Interpretation/ scientific implications**	17	a. Interpret the results, taking into account the study objectives and hypotheses, current theory and other relevant studies in the literature.	The study confirms that Ar significantly reduced tumor load, validating our primary hypothesis. Its effect is likely mediated by beneficial metabolites.
		b. Comment on the study limitations including potential sources of bias, limitations of the animal model, and imprecision associated with the results.	The exact host pathways responsible for the observed tumor-suppressing effect remain unknown.
**Generalisability/ translation**	18	Comment on whether, and how, the findings of this study are likely to generalise to other species or experimental conditions, including any relevance to human biology (where appropriate).	The results in vrtro and vivo underscores the generalizable nature of Ar's tummor suppressive effect.
**Protocol registration**	19	Provide a statement indicating whether a protocol (including the research question, key design features, and analysis plan) was prepared before the study, and if and where this protocol was registered.	A formal protocol detailing the research question, design, and analysis plan was established before the commencement of the study, which was used to obtain the animal ethics. This protocol was not registered.
**Data access**	20	Provide a statement describing if and where study data are available.	
**Declaration of interests**	21	a. Declare any potential conflicts of interest, including financial and non-financial. If none exist, this should be stated.	NA
		b. List all funding sources (including grant identifier) and the role of the funder(s) in the design, analysis and reporting of the study.	GRF, Hong Kong (14104523), CRF, Hong Kong (C7042-21G), and Midstream Research Programme for Universities, ITF, Hong Kong (MRP/058/20).The funders had no role in the design, analysis and reporting
